# Protecting tropical forests from the rapid expansion of rubber using carbon payments

**DOI:** 10.1038/s41467-018-03287-9

**Published:** 2018-03-02

**Authors:** Eleanor M. Warren-Thomas, David P. Edwards, Daniel P. Bebber, Phourin Chhang, Alex N. Diment, Tom D. Evans, Frances H. Lambrick, James F. Maxwell, Menghor Nut, Hannah J. O’Kelly, Ida Theilade, Paul M. Dolman

**Affiliations:** 1 School of Environmental Sciences, University of East Anglia, Norwich, NR4 7TJ UK; 2 Department of Biology, University of York, York, YO10 1DD UK; 3 Department of Animal and Plant Sciences, University of Sheffield, Sheffield, S10 2TN UK; 4 Department of Biosciences, University of Exeter, Stocker Road, Exeter, EX4 4QD UK; 5 Forest and Wildlife Research Institute, Forestry Administration, Royal Government of Cambodia, Hanoi Street 1019, Phum Rongchak, Sankat Phnom Penh Tmei, Khan Sen Sok, Phnom Penh, 12010 Cambodia; 6 Wildlife Conservation Society Cambodia Program, Street 21, Sangkat Tonle Bassac, Khan Chamkarmorn, Phnom Penh, 12300 Cambodia; 7 Wildlife Conservation Society Global Conservation Program, 2300 Southern Boulevard, Bronx, NY 10460 USA; 8 Department of Plant Sciences, University of Oxford, South Parks Road, Oxford, OX1 3RB UK; 9 Department of Food and Resource Economics, University of Copenhagen, Rolighedsvej 25,, DK-1958 FrbC Denmark; 10 Forestry Administration, Royal Government of Cambodia, 40 Preah Norodom Boulevard, Phnom Penh, 12205 Cambodia

## Abstract

Expansion of *Hevea brasiliensis* rubber plantations is a resurgent driver of deforestation, carbon emissions, and biodiversity loss in Southeast Asia. Southeast Asian rubber extent is massive, equivalent to 67% of oil palm, with rapid further expansion predicted. Results-based carbon finance could dis-incentivise forest conversion to rubber, but efficacy will be limited unless payments match, or at least approach, the costs of avoided deforestation. These include opportunity costs (timber and rubber profits), plus carbon finance scheme setup (transaction) and implementation costs. Using comprehensive Cambodian forest data, exploring scenarios of selective logging and conversion, and assuming land-use choice is based on net present value, we find that carbon prices of $30–$51 per tCO_2_ are needed to break even against costs, higher than those currently paid on carbon markets or through carbon funds. To defend forests from rubber, either carbon prices must be increased, or other strategies are needed, such as corporate zero-deforestation pledges, and governmental regulation and enforcement of forest protection.

## Abstract (web summary)

Expansion of rubber plantations threatens tropical forest carbon stocks and biodiversity, but may be dis-incentivised using carbon finance. Here, Warren-Thomas et al. use forest and agricultural data for Cambodia to show that carbon prices of $30–$51 per tCO_2_ are needed to match forest protection costs.

## Introduction

Forest is being converted to *Hevea brasiliensis* rubber plantations across Southeast Asia, resulting in the loss of forest carbon stocks and substantial declines in biodiversity^1–3^. Eighty five per cent of global rubber area occurs in Southeast Asia, where expansion has driven northward into Cambodia, China, Laos, Myanmar and Vietnam (hereafter termed mainland Southeast Asia, but also known as the Indo-Burma biodiversity hotspot), replacing forest and traditional swidden cultivation^1,2^. Despite its massive extent (8.6 million ha in Southeast Asia in 2014, equivalent to 67% of oil palm extent^4^) and comparable negative consequences for biodiversity and ecosystem services^5^, conversion of forest to rubber monoculture has not faced the same public scrutiny as oil palm. Here, we analyse carbon outcomes and opportunity costs of forgoing forest conversion to rubber, including profits from timber extraction, and ask whether permitting selective logging could reduce these opportunity costs to improve the likelihood of success for forest carbon finance.

Where climatic conditions are suitable for both oil palm and rubber, they can generate similar profits per unit land area, but oil palm provides better returns if labour supply is restricted^5^. However, rubber can tolerate a wider range of climatic conditions and soil types, permitting its expansion into mainland Southeast Asia^2^, although reduced yields and tree mortality are reported from many northern parts of its range^1^. Recent expansion of rubber has mostly occurred in areas unsuitable for oil palm^1,6^.

Demand for natural rubber continues to grow, predominantly driven by the tyre industry, and plantations are predicted to expand by 4.3–8.5 million ha within a decade^2^. This expansion not only threatens forest carbon stocks, but also has serious implications for biodiversity conservation. The forests of mainland Southeast Asia are globally unique ecosystems, supporting numerous threatened and endemic animal, bird, invertebrate and plant species^7,8^, including exceptionally valuable luxury timbers (eg, rosewoods, *Dalbergia* spp.).

Rubber prices are currently relatively low (Supplementary Fig. 1), offering a lull in expansion, and an opportunity to develop strategies for future planting that minimise negative outcomes for climate and forests. Stemming rubber expansion onto biodiversity-rich forest could reduce carbon emissions and achieve conservation gains simultaneously, making efficient use of limited funds^9,10^. However, the effectiveness of any forest carbon finance scheme will depend on the number of carbon credits generated, the perceived costs of conserving forest and the price offered for carbon credits.

Costs of forest conservation can be broken down into practical costs of conservation activities (transaction and implementation costs) and the costs of forgone economic activity (opportunity costs); these are borne at local, sub-national or national scales. In contrast, the economic costs of climate change are largely borne globally. Estimates of damage costs caused by carbon emissions are termed the “social cost of carbon”, and are used in the design of policies to regulate carbon emissions^11^. Such estimates have been made using various methodologies, and vary by an order of magnitude^11–16^, but perhaps the most policy-relevant is that developed for the government of the United States ($36 per tCO_2_ in 2015^11,13,17^), which we use to discuss the context of our results.

**Fig. 1:**
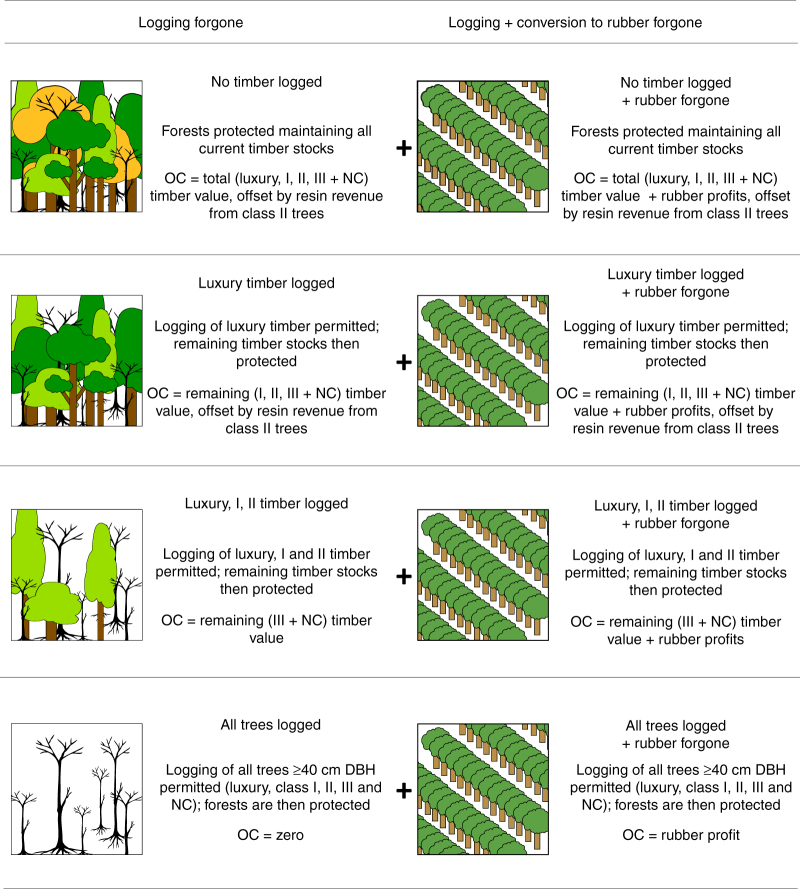
Scenarios of intervention to protect forest from conversion to rubber. Schematic shows scenarios of intervention to protect remaining forest in: initial state (“No timber logged”); after selective logging (“Luxury timber logged” or “Luxury, I, II timber logged”) and after all trees (≥40 cm DBH) have been logged (“All trees logged”). Tree species are assigned to one of four royalty classes: luxury, I, II, III or non-classified (NC; Supplementary Data 1) based on a governmental list^69^; collection of luxury timber includes all stems ≥10 cm DBH, for other classes stems ≥40 cm DBH. “Timber” refers to luxury, class I and class II species; class III and NC species are non-commercial, and are thus termed “trees”. All resin trees are classified as class II. The left column considers opportunity costs (OC) of protecting forests following scenarios of logging only, where conversion to rubber is prohibited; the right column considers opportunity costs of protecting forests following scenarios of logging, with the additional opportunity costs of forgone conversion to rubber

We analysed the carbon outcomes and opportunity costs of forgoing forest conversion to rubber, including profits from timber extraction, and asked whether permitting selective logging could reduce opportunity costs and improve the likelihood of success for forest carbon finance. We modelled scenarios of protecting either intact forest (“No timber logged”), or forest degraded by permitting selective logging (felling only trees ≥40 cm DBH at three intensity levels, depending on timber royalty/value classes: (1) “Luxury logged”; (2) “Luxury, I, II logged”; (3) “All trees logged”), from subsequent conversion to rubber (Fig. 1; Supplementary Note 1). We calculated rubber profits based on typical monocultural plantation systems, containing high-yielding clonal varieties of rubber planted at densities of 400–550 stems per ha^18^. Such systems are ubiquitous across mainland Southeast Asia, within both smallholdings and larger estates^1,19–21^. We also included the economic value of dipterocarp tree resin collection as an economic benefit of forests retaining class I and II timber species (all resin species are class II). As future market changes could influence the relative profitability of alternative crops, we also considered potential profits from other cash crops in Cambodia: cassava, cashew, and sugarcane. For each scenario, we estimated the breakeven carbon price ($ per tCO_2_) that would match the opportunity costs of forgoing further logging and conversion to rubber, plus the transaction and implementation costs of a carbon finance scheme. We used a substantial inventory data of 20,281 trees (DBH ≥10 cm; Supplementary Table 1) from six forest landscapes in Cambodia widely spaced across ~260 km of dense and open forest (Supplementary Fig. 2) to make highly robust estimates of forest carbon stock and timber volumes.

These span two forest types in zones climatically suitable for rubber^1^: “dense” evergreen and semi-evergreen (35–55 m height) and “open” deciduous/mixed-deciduous forests (25–35 m)^22^. They include the largest remaining contiguous lowland evergreen forest in mainland Southeast Asia^23^ and globally significant extents of open forest^24^.

Using cost benefit analysis and net present value, we find that the carbon prices needed to match the costs of protecting intact or selectively-logged forests in mainland Southeast Asia from conversion to rubber are $29.86–$37.48 per tCO_2_ for dense forests, or $30.93–$51.11 per tCO_2_ for open forests. Rubber forms a far greater proportion of opportunity costs than timber, and breakeven carbon prices are much lower under scenarios of logging alone. Our calculated prices are higher than those currently paid on carbon markets (~$5–$13 per tCO_2_), or through carbon funds (~$5 per tCO_2_), but are close to, or below, policy-relevant estimates of the social cost of carbon ($36 per tCO_2_). The mismatch between predicted social costs of carbon at the global scale, carbon market prices and the local costs of avoiding carbon emissions from deforestation, poses a serious challenge for forest carbon finance in tackling global climate change. Given our findings, additional strategies beyond carbon finance will be needed to prevent forest conversion to rubber. However, risk aversion and non-economic values of forest could reduce the carbon payments needed to leverage protection, relative to our calculations.

## Results

### Timber volume and carbon stocks

Harvestable wood volume of all tree species, assuming a minimum harvestable DBH of 40 cm (≥10 cm for luxury timber; Supplementary Note 1), was 49.4 ± 0.5 m^3^ per ha in dense forest, but just 13.6 ± 0.3 m^3^ per ha in open forest (mean ± SE; Table 1). Timber of royalty classes I and II accounted for 54% of volume in dense forest and 69% in open forest. Luxury timber was rare, contributing only 1.1 ± 0.0 m^3^ per ha in dense forest (2%) and 1.3 ± 0.1 m^3^ per ha in open forest (10%).

**Table 1:** Mean carbon stock and wood volume held in harvestable stems of each timber royalty class in dense and open forests

Forest type	Timber royalty class	Carbon stock ≥40 cm DBH (tC per ha)	Carbon stock ≥30 cm DBH (tC per ha)	Wood volume ≥40 cm DBH (m^3^ per ha)	Wood volume ≥30 cm DBH (m^3^ per ha)
Dense	Luxury	2.2 ± 0.0		1.1 ± 0.3	
	I	30.8 ± 0.4	35.0 ± 0.4	16.9 ± 0.2	19.2 ± 0.3
	II	12.1 ± 0.3	14.6 ± 0.3	9.8 ± 0.2	12.1 ± 0.2
	III	3.5 ± 0.1	5.3 ± 0.1	2.4 ± 0.1	3.4 ± 0.1
	Non-classified	39.0 ± 0.4	53.5 ± 0.5	19.3 ± 0.2	26.2 ± 0.2
Open	Luxury	1.8 ± 0.1		1.3 ± 0.1	
	I	20.5 ± 0.5	29.5 ± 0.1	6.6 ± 0.1	14.7 ± 0.3
	II	4.6 ± 0.1	9.1 ± 0.0	2.7 ± 0.1	7.0 ± 0.1
	III	0.5 ± 0.0	1.2 ± 0.4	0.3 ± 0.0	1.5 ± 0.0
	Non-classified	8.0 ± 0.3	12.6 ± 0.1	2.7 ± 0.1	8.0 ± 0.2

Mean carbon stock and wood volume are shown with the 95% confidence interval of the mean

Mean forest carbon stocks, measured as combined above-ground and below-ground biomass (AGB and BGB) of all stems ≥10 cm DBH, were 194 ± 1.2 tC per ha in dense forest (123–284 tC per ha among individual landscapes) and 104 ± 0.8 tC per ha in open forest (60–157 tC per ha among landscapes; Table 1, Supplementary Fig. 3). Lower carbon stocks in open forest reflected both a greater proportion of smaller stems (Supplementary Fig. 4) and lower stem density (mean across inventories 213–311 per ha in open; 415–589 per ha in dense; Supplementary Data 1), despite similar wood density (weighted mean 0.713 g cm^−3^ in open; 0.630 g cm^−3^ in dense).

**Fig. 2:**
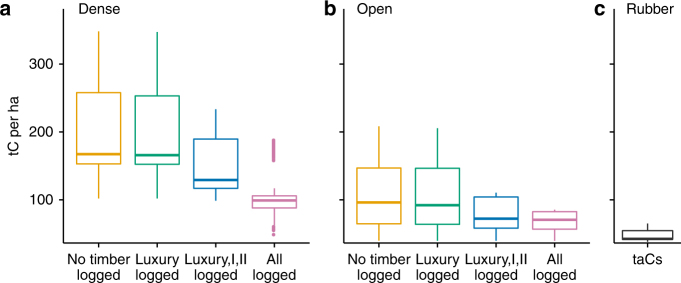
Effects of selective logging on dense and open forest carbon stocks. Carbon stock (tC per ha) of **a** dense and **b** open forest in initial state (“No timber logged”) and under scenarios of selective or complete logging (following Fig. 1), and **c** time-averaged carbon stocks (taCs) of rubber plantations in Southeast Asia^3^. Central bar shows median, box shows upper and lower quartiles, whiskers extend to 1.5× the inter-quartile range, and outliers are presented as dots

Forest carbon stock changed minimally following selective logging of luxury timber (“Luxury logged” scenario), reducing by 1% in dense forest and 2% in open. Additional logging of classes I and II (“Luxury, I, II logged”) reduced carbon stocks by 20% in dense forest (to 175 ± 1.1 tC per ha) and 26% in open forest (to 78 ± 0.7 tC per ha; Table 1; Fig. 2). Even the removal of all trees ≥40 cm DBH (“All trees logged”), left 60% (dense), and 66% (open) of original carbon stock. Therefore, substantial forest carbon is retained before conversion to rubber, even after logging of valuable timber or removing all large trees.

Rubber plantations are considered forest cover in FAO Forest Resources Assessments^25^, while the USAID LEAF Atlas maps rubber-dominated landscapes as forest (such as Southern Thailand), but protected open forests in Eastern Cambodia as non-forest^26^. However, we find that even assuming high post-deforestation time-averaged carbon stocks (taCs) for rubber of 52.5 tC per ha (from Cambodia, Thailand and Indonesia^3^, likely greater than will be achieved in seasonal open forest environments), conversion of intact forest to rubber would still generate net losses of 141.5 ± 1.2 tC per ha in dense forest and 51.5 ± 0.8 tC per ha in open forest (Fig. 2). Even conversion of degraded logged open forest would generate net emissions, as well as biodiversity loss. Additionally, although we do not account for changes in soil organic carbon (SOC) because the Intergovernmental Panel on Climate Change (IPCC) tier 1 carbon outcome calculation method assumes no SOC change with conversion to perennial tree crops, conversion of lowland forest to rubber plantations does generate SOC emissions^27^, which, if included, would increase net emissions.

### Logging and conversion to rubber

**Fig. 3:**
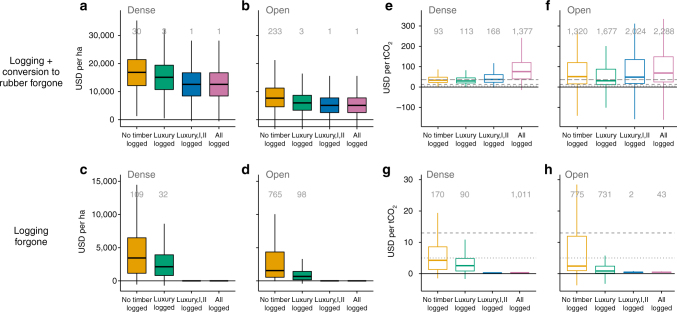
Opportunity costs and breakeven carbon prices needed to protect forests from logging and conversion to rubber. Opportunity costs **a**–**d** include forgone profits from logging and rubber, or logging alone, offset by resin revenue except where resin trees (all class II) are logged out (in the “Luxury, I, II logged” and “All trees logged” scenarios). Breakeven carbon prices **e**–**h** are the prices needed to offset combined opportunity costs and carbon finance scheme setup (transaction) and implementation costs. Costs are shown separately for dense and open forests. Time-averaged post-deforestation land-use carbon stocks (taCs) partially offset forest carbon losses. Scenarios (of permitted selective logging, with and without potential for conversion to rubber) follow Fig. 1; boxplot format as Fig. 2. Grey lines on breakeven carbon price panels **e**–**h** represent indicative global carbon prices: dotted = $5 per tCO_2_ (voluntary market forest carbon sales and non-market carbon fund prices), short dash = $13 per tCO_2_ (compliance market prices) and long dash = $36 per tCO_2_ (social cost of carbon, not shown on “Logging forgone” plots; see Supplementary Table 4). Outliers (>1.5× the interquartile range) are not displayed to improve the clarity of the figure; the value shown above each box-whisker gives the *n* outliers excluded out of 10,000 modelled results

Total opportunity costs of protecting intact forest (“No timber logged + forgone rubber” scenario), calculated as the profit from a single offtake of all commercial timber (Supplementary Table 2) plus the 25-year net present value (NPV; 10% discount rate) from subsequent rubber plantations (Supplementary Table 3), were $16,841 (median, interquartile range $12,118–$21,397) per ha in dense forest and $7,674 ($4,581–$11,250) per ha in open forest (Fig. 3). Intervening after removal of luxury timber (“Luxury timber logged + forgone rubber” scenario) reduced opportunity costs of logging by 38% in dense forest and 56% in open; however, total opportunity costs (including rubber) were only reduced to $15,097 ($10,738–$19,390) per ha and $5,956 ($3,341–$8,663) per ha, respectively. After all valuable timber had been logged out (“Luxury, I, II, logged” or “All trees logged”), the opportunity cost of rubber alone was $12,570 ($8,436–$16,698) per ha in dense forest, and $5,089 ($2,532-$7,764) per ha in open forest. Rubber therefore forms the majority of total opportunity costs (75% of median in dense forest, 66% in open forest). The substantially lower NPV of rubber in open forest arises from the delay of tapping from 6 years to 10 years after planting, due to slower tree growth in drier conditions. Using a discount rate of 8% to allow comparison, our estimate of rubber NPV in dense forest areas ($16,533 per ha, $11,403–$21,732 per ha) is similar to estimates from lowland Xishuangbanna, China (~$19,800 per ha, 25-year NPV, 8% discount rate)^21^.

**Fig. 4:**
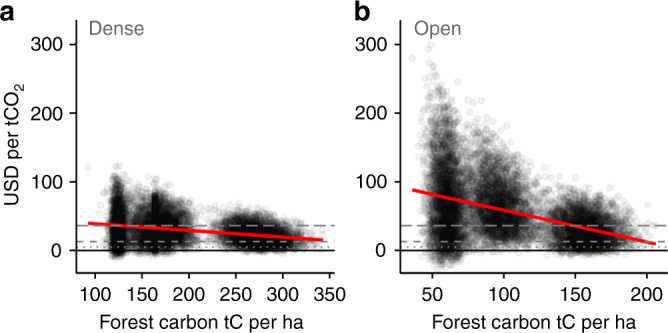
Effect of forest carbon stock on breakeven carbon prices in dense and open forests. The response of breakeven carbon price to forest carbon stock under the “No timber logged” scenario is shown for **a** dense and **b** open forests. Each dot represents the outcome of one model iteration. Grey dashed lines indicate real-world carbon prices, following Fig. 3. Red lines represent a linear model relating forest carbon to breakeven carbon price (with grey shaded SE too narrow to be visible)

High-opportunity costs translated into high breakeven carbon prices, far greater than indicative carbon prices currently paid in voluntary carbon market sales and carbon funds ($5 per tCO_2_) or compliance market sales ($13 per tCO_2_), although for dense forests, breakeven prices were below the estimated social cost of carbon ($36 per tCO_2_; Supplementary Table 4). Protecting intact forest (“No timber logged + forgone rubber”) required $33.43 (median, interquartile range $22.65–$48.20) per tCO_2_ for dense forest and $51.11 ($15.59–$120.19) per tCO_2_ for open forest (Fig. 3). Removal of luxury timber reduced breakeven prices to $29.86 ($20.02–$44.96) per tCO_2_ in dense forest and $30.93 ($11.95–$87.78) per tCO_2_ in open forest, bringing the latter below the estimated social cost. This was because luxury timber comprised only a small proportion of forest carbon (Table 1), but a large proportion of timber value (Supplementary Table 2), so that logging opportunity costs were substantially reduced while forest carbon stocks remained mostly intact. Although further logging of all valuable timber (“Luxury, I, II logged + rubber”) reduced the opportunity costs of logging to zero, breakeven prices in this scenario were actually higher than the intact forest scenario for dense forest ($37.48, $23.28–$60.90 per tCO_2_), and reduced only slightly in open forest ($48.83, $17.56–$135.18 per tCO_2_). This was due to the large proportion of forest carbon held in class I and II species (Table 1); removing large trees of these timber classes substantially depleted residual carbon stocks while reducing logging opportunity costs. With fewer carbon credits a greater breakeven price was needed to offset the opportunity cost of rubber. In a few model iterations, conversion of heavily degraded open forest to rubber generated net carbon gains, producing negative carbon prices. This effect is further shown in Fig. 4; although variation in opportunity costs leads to substantial variation in carbon prices, in both forest types, prices also decrease as forest carbon stocks increase.

### Sensitivity analysis

**Fig. 5:**
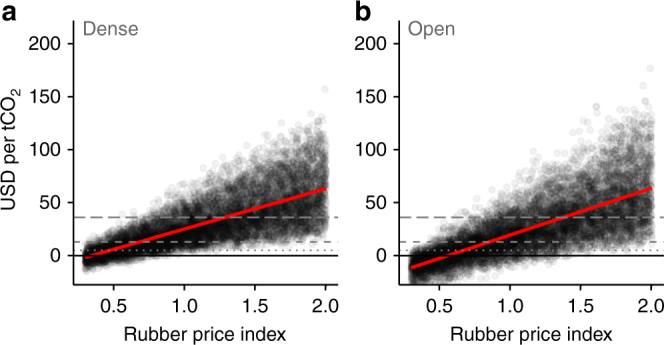
Effect of rubber price on breakeven carbon prices in dense and open forests. The response of breakeven carbon price to rubber prices under the “No timber logged” scenario is shown for **a** dense and **b** open forests. Each dot represents the outcome of one model iteration. Grey dashed lines indicate real-world carbon prices, following Fig. 3. Red lines represent a linear model relating breakeven carbon price to rubber price index (with grey shaded SE, too narrow to be visible), where an index value of 1.0 is the 10-year mean rubber price (2003–2012)

Farm gate prices for rubber strongly influenced carbon breakeven prices in both dense and open forest (Fig. 5). To make our results robust to short-term price volatility (Supplementary Fig. 1), we used a 10-year mean price (2003–2012; $2,595 ± 200 per t). However, even using the relatively low 2014 rubber price ($1,644 ± 200 per t, indexed at ~0.6 relative to the 10-year mean in Fig. 5), breakeven prices in the “No timber logged + forgone rubber” scenario only reduced to $19.09 ($11.52–$27.82) per tCO_2_ in dense forest, and $16.08 ($0.14–$60.65) per tCO_2_ in open forest, still higher than carbon market prices, although well below the estimated social cost.

The NPVs of three alternative cash crops (cassava, cashew and sugarcane) were also considered, to account for potential future changes in relative profitability or demand for agricultural commodities. Oil palm was not assessed as much of mainland Southeast Asia is marginal or unsuitable for its cultivation^6^; however, 25-year oil palm NPV in Malaysia was estimated to be $11,240 per ha^28^. Estimates of cassava NPV exceeded that of rubber ($14,597, $10,133–$19,124 per ha), but other crops were less profitable (Supplementary Table 3). We note that cassava yields and prices could be lower than our estimates, due to the potential for nutrient depletion with repeated cultivation and lack of access to markets, which we have not accounted for (Supplementary Table 5). Although all three crops store less carbon than rubber (Supplementary Table 6), cassava and cashew still generated high breakeven carbon prices in the “No timber logged” scenarios: for cassava $27.02 ($19.59–$36.66) per tCO_2_ in dense forest and $49.76 ($31.54–$76.14) per tCO_2_ in open forest, and for cashew $21.98 ($15.29–$30.31) per tCO_2_ and $47.21 ($25.59–$75.52) per tCO_2_, respectively (Supplementary Fig. 5). Breakeven prices were affected by crop price, as for rubber (Supplementary Fig. 6), but omitting potential revenues from dipterocarp resin harvest (Supplementary Table 7), and changing resin prices (Supplementary Fig. 6), had negligible effects on breakeven prices.

Predicted NPVs of all crops were highly sensitive to changes in discount rate. Rubber NPV increased to ~$25,800 per ha with a 5% discount rate in dense forest (giving a breakeven carbon price of $60.04, $41.20–$88.06 per tCO_2 _assuming protection of an intact forest) and $14,700 per ha in open forest ($108.68, $43.43–$255.92 per tCO_2_). A 15% discount rate reduced rubber NPV to ~$6,200 per ha in dense forest ($20.62, $13.60–$29.41 per tCO_2_) and $1,100 per ha in open forest ($20.12, $3.47–$62.34 per tCO_2_; Supplementary Table 3 and Figs. 7, 8). We use 10% for our main analysis to allow comparability with other studies, but the choice of discount rate introduces substantial variation in our estimates of opportunity cost. Discount rates of 5% are commonly used in social investments^29^, but discount rates of 8% have been recommended for cost-benefit analysis in Asia^21^; we therefore present the full range of NPV results in Supplementary Table 3.

### Logging without conversion to rubber

If the threat of forest conversion to rubber could be removed, our analysis shows that carbon prices close to those currently paid on voluntary markets and carbon funds ($5 per tCO_2_) could meet the opportunity costs of logging in mainland Southeast Asia. This could be achieved through governmental land-use planning or zoning, market exclusion of “deforestation rubber” via development of a robust sustainability initiative^30^, or further corporate zero deforestation commitments such as that announced by Michelin^31^. In this case, opportunity costs of forgoing logging (“No timber logged”) were less than a quarter ($3,443, $1,151–$6,490 per ha) in dense forest and around a fifth ($1,534, $563–$4,356 per ha) in open forest (Fig. 3, Supplementary Table 2) of the costs of forgoing both logging and rubber in the “No timber logged + rubber forgone” scenario. Consequently, if forest conversion is prevented by other means, the median breakeven carbon price of protecting intact dense forest from any logging was only $4.27 ($1.33–$8.56) per tCO_2_, and $2.43 ($0.95–$11.95) per tCO_2_ in open (Fig. 3).

Changes in timber price influenced breakeven carbon prices in logging-only scenarios (Supplementary Fig. 6). Likewise, reducing the minimum threshold for timber harvest to 30 cm DBH increased breakeven carbon prices of intact forest to $5.23 ($2.23–$9.61) per tCO_2_ in dense forest and $6.26 ($3.52–$16.22) per tCO_2_ in open forest, with the opportunity cost of logging in open forests more than doubling to $3,373 ($1,214–$6,346) per ha, owing to the high density of class I and II stems 30–40 cm DBH in open forest (Supplementary Table 2).

## Discussion

Our findings show that forest conversion to rubber may currently generate far more revenue than a carbon finance scheme, but that profits from logging are sufficiently low for carbon finance to be a competitive alternative when rubber is not a threat. Under current market conditions, avoiding deforestation for rubber thus requires recognition of environmental, social or other ecosystem service benefits of forests by governments, and a willingness to accept apparent economic costs. However, for dense forests, mean breakeven prices were closely aligned to estimates of the social cost of carbon, and when considering relatively low rubber prices, breakeven carbon prices for both forest types were substantially below the social cost. This makes forest conversion to rubber a poor option from a global perspective on cost-effective climate change mitigation.

We used a conservative value of $36 per tCO_2_ for the social cost of carbon, based on estimates developed for and used by the government of the United States^13,17^. In contrast, a 2011 review calculated a mean of $177 per tCO_2_ (SD $293 per tCO_2_)^16^ and subsequent analyses gave estimates of $103–$138 per tCO_2_^14^ or $220 per tCO_2_^15^. Considering these higher estimates, preventing forest conversion to rubber represents cost-effective action to reduce emissions, even for intact open forests. However, the social cost of carbon is based on the premise of a global cost-benefit exercise, comparing the economic cost of carbon emissions to the cost of controlling emissions^12^. A key issue is that, even where these cost estimates align, in the absence of a global carbon pricing mechanism the path from a calculated social cost of carbon to compensation for the local-scale costs of avoided emissions is unclear.

There are indications that carbon market prices could be raised much closer to the social cost of carbon in some sectors to meet climate change targets: for instance, if recent proposals to set a price floor of €20–€30 (~$23–$34) per tCO_2_ on the EU Emissions Trading Scheme come to fruition, a new global benchmark could be set^32^. Recent research investigating the incentives required to decarbonise the energy sector to meet the Paris Agreement targets (i.e., limiting global temperature rise to below 2 ^o^C) similarly found the need to introduce carbon prices to the industry and power sectors of all countries, with prices of $20 per tCO_2_ by 2020 and $120 per tCO_2_ by 2030 in OECD countries, $10–$90 per tCO_2_ in major emerging economies, and $5–$30 per tCO_2_ elsewhere^33^. Carbon finance for dense forests could then be nearly as lucrative as conversion to rubber in the near-term, while in the longer-term, prices could be high enough to defend even open forests, notwithstanding the risk of unintended market feedbacks arising from restricted supply (see below)^34^. We also note that even if full opportunity costs of conservation are not met, smaller financial incentives to conserve forest may be an attractive option if there is existing social or political pressure to conserve, or where non-market values are recognised.

Open forests tend to be drier with poorer sandier soils^35^ and thus may have lower agricultural potential. We incorporated the effect of delayed maturation of rubber under drier open forest conditions, but robust data on rubber yields in such conditions are notably lacking^36^. Poorer growth could also result in lower rubber carbon stocks^1,3^, reducing the breakeven price needed to match rubber profits in open forest areas, and improving the prospects for protection using carbon finance. Robust data on the relative yield and carbon stocks of rubber plantations established on former open or dense forests are urgently required^3^. However, where land proves unfavourable for rubber, the high potential NPV of cassava, known to grow successfully in less favourable environments, at least in the short term (Supplementary Table 5), reinforces the importance of other mechanisms to curb forest conversion.

Although rapid expansion of cassava for animal feed contributed to serious deforestation of northern Thailand in the late 20th century^37^, and in Cambodia cassava expanded at a similar rate to rubber within the last decade (see Methods), in the longer-term, cassava extent has been relatively stable across Asia (3.9 million ha in 1981, 4.1 million ha in 2014), although yields nearly doubled (11.9 t per ha per year in 1981, 21.9 t per ha per year in 2014^38^). This contrasts strongly with Asian rubber extent, which almost doubled from 5.1 million ha in 1981 to 9.8 million ha in 2014, despite similar yield increases (0.7 t per ha per year in 1981, 1.2 t per ha per year in 2014)^38^. Thus, although we estimate median NPV to be greater for cassava than rubber, demand does not currently appear to be driving expansion of cassava onto forest to the same degree as rubber in Asia. In addition, Cambodian cassava growers currently face problems accessing markets and supply chains, and depletion of soil quality and declining yields with repeated cultivation are known issues^39^. We acknowledge that this may reduce the cassava NPV realised by farmers relative to our estimates, although we have been unable to quantify this (Supplementary Table 5). Nevertheless, use as a biofuel feedstock may further increase cassava demand within Asia and at the global level it is rapidly expanding^40^. Together with our high estimated NPV for cassava this suggests that, although not currently a major regional driver of deforestation in Asia, the importance of cassava may increase.

We have used a non-spatial approach to estimate breakeven carbon prices for dense and open forest in mainland Southeast Asia. We show that rubber NPV is likely substantially lower in open than in dense forest areas, and were able to incorporate geographic variability in timber and carbon stocks, which influenced breakeven carbon prices. Additional spatial variation in rubber NPV and carbon breakeven prices will be generated by distance to markets, regional farm gate prices, yields (affected by soil type and quality, topography, elevation, water availability, planting material, etc.), and labour costs, amongst others. However, spatially explicit data on potential rubber NPV are not currently available for most of mainland Southeast Asia, including Cambodia, not least because recently established plantations are not yet productive. This precluded meaningful marginal cost curve analyses to quantify the additional area of rubber expansion that could potentially be avoided with incremental increases in carbon price. Such spatial analysis has been conducted locally for Xishuangbanna, a hotspot of rubber expansion in Southwest China, where field data from existing plantations show that NPV decreases with greater elevation, particularly above 900 m asl^21,41^. Once context-specific rubber yield, price and cost data become available, marginal cost analyses may be possible for mainland Southeast Asia. Nevertheless, if carbon finance focuses on the cheapest avoided emissions first, making each additional tonne of carbon more expensive than the last, initial efforts to protect forests from rubber might only require prices at the low end of our presented range. However, if forests have good potential for conversion to rubber but are also priorities for conservation using carbon finance, due to high carbon densities and/or biodiversity value, costs may be much higher.

While we have assessed the likelihood of success for carbon finance schemes in tackling forest conversion to rubber under current conditions, and considered a range of potential price changes, rubber prices may rise in the future if rubber production is constrained relative to demand due to restriction of planting in forested areas^34^. The design of any initiative attempting to prevent forest conversion to rubber would therefore need to account for potential market feedbacks generated through conservation activities, which could otherwise generate net negative outcomes for carbon emissions and biodiversity^34^. Ultimately, the demand for natural rubber might only be mitigated through further development of synthetic alternatives (although these are currently petroleum based and may represent a worse outcome for carbon emissions), or though improvements in methods for recycling natural rubber.

We compared mutually exclusive management options, for a hypothetical hectare of forested land, using a cost benefit analysis framework based on a single metric, NPV. This approach assumes that the single land use with the highest NPV will be preferred, and that if carbon prices match this NPV (together with the transaction and implementation costs of protecting forest), then forest protection will be the preferred option. However, as this does not incorporate other influences on land-use decisions, such as variability in future returns (uncertainty, used here synonymously with risk^42^), it provides only an approximation of the costs of forest protection, and the level of carbon finance payments needed to influence decision-making.

Decision makers, including low-income farmers in the tropics, tend to be risk averse^43^. A key strategy for reducing risk is to diversify income sources by establishing a portfolio of land uses, that provides income resilience despite lower financial returns, for instance by growing a range of crops with independently fluctuating yields^42,43^. Alternative approaches to economic analysis of PES schemes, that incorporate risk management and the option to assess multiple land uses simultaneously, include “Optimised Land-Use Diversification”, which considers portfolios of multiple land uses, returns, and risk^42^, and stochastic dominance, with^44^ or without^45^ land use portfolios. Portfolio theory^46^ has also been used to assess decision making around a variety of natural resource and land use problems^47^.

Understanding the likely outcome of risk aversion in the context of rubber expansion can be considered within two broad scenarios: first, planting in large estates by companies (as in this analysis), and second, planting by smallholder farmers. In the first scenario, a company may already have a diverse portfolio of land holdings (each covering hundreds or thousands of hectares) across multiple locations, of which a rubber plantation could form a single component. This scenario is relevant for Cambodia, where large tracts of forested land are allocated for specific, single, purposes (Economic Land Concessions for a rubber plantation^48^, or establishment of a protected area). We consider diversification within a single large land holding unlikely in the Cambodian context. Furthermore, carbon finance schemes in Cambodia have so far been established to avoid deforestation of large intact forest areas^22^. Thus, in the context of this study, assumptions underlying alternative modelling approaches such as Portfolio Theory may not be strongly relevant.

In the second scenario, a smallholder may split a handful of hectares of land among multiple crops (cash or subsistence); particularly relevant in countries where rubber production on much smaller land holdings is the norm (such as Thailand or China^2^). Risk-averse behaviour by such farmers could favour the uptake of PES schemes if they are perceived to offer stable income, although opportunity costs remain key^43^. However, the outcome of incorporating risk aversion into calculation of PES payments in the context of smallholder farmers is variable. Models of forest conversion in South America that incorporated risk aversion reduced predicted carbon payments (needed to offset opportunity costs) by more than half relative to models that ignored risk; incorporating a portfolio of land uses, price-supply relationships, demand shifts and agricultural intensification also reduced predicted carbon payments relative to solely considering compensation for the single highest-value land use^42^. In contrast, conservation payment levels required to retain shade coffee versus conversion to maize in Ecuador increased up to twofold when accounting for risk aversion, due to higher revenue variability for coffee than maize^44^, but subsequent analysis that incorporated plantain revenue (planted within coffee agroforests), reversed this pattern^49^. Application of alternative modelling approaches may thus be particularly valuable in the context of smallholder rubber farms.

Additional caveats to our findings are that we assume a single land use is established and maintained for a 25-year period; while this assumption is likely to hold for rubber plantations that are typically managed on a 25-year rotation^18^, this is less likely for shorter rotation crops such as cashew (10 years) or cassava (one year). Our cost benefit analysis also ignores the near irreversibility of deforestation (compared to the relative ease of switching between alternative agricultural land uses), which may reduce required compensation payments^45^.

As with the other approaches discussed here, the use of NPV fails to capture the full utility value of alternatives, including non-carbon ecosystem services, health or cultural benefits of retaining forests, or the impacts of agricultural conversion on water quality, soils, or food security. Individual farmers’ decisions might also be influenced by governmental policies, regulations, land and climatic conditions, gender, demography, and values^43^. Multi-criteria decision-making models are another alternative analytical approach, which could incorporate such non-market considerations together with risk^50^. Lastly, we recognise that decisions about participating in a carbon finance scheme, such as REDD+, involve an additional and specific suite of considerations^51^, including internationally agreed targets and commitments to halt climate change.

While our results suggest there are major impediments to the use of carbon financing in halting rubber expansion, they show that selective logging alone could be prevented by such payments. In contrast to the timber volumes of mainland Southeast Asia’s dense forests calculated in this study, timber volumes in forests of insular Southeast Asia are substantially higher (84.9 ± 9.0 SE per m^3^ per ha, 50–60 cm DBH^52^). This translates into higher timber profits ($5,563 ± 757 SE per ha in dense forest^52^), requiring higher carbon prices to match the opportunity costs of logging (eg, $22–$28 per tCO_2_^28^) than on the mainland. However, despite the substantial and robust forest inventory dataset that underpins this study, and the relatively low estimated timber profits compared to rubber cultivation, the presence of high-value luxury timbers^53^ poses a distinct problem for timber valuation in mainland Southeast Asia. Timber prices, particularly for luxury species, increase up the supply chain (Supplementary Table 8) and the Cambodian timber trade is highly opaque, involving informal payment systems and considerable illegality^48,53^. Such issues are found across mainland Southeast Asia. Although using local-level timber prices was appropriate due to the lack of formal timber markets, this underestimates total opportunity costs accruing to hidden but powerful actors, especially those selling timber illegally on international markets. These additional opportunity costs may play out through pressure on governmental decision makers, particularly those involved in land allocation. Thus, forest conservation efforts based on climate change or biodiversity outcomes cannot, in this context, be divorced from social demands for governance and accountability^48^.

In the context of current carbon finance markets and funds for REDD+, policy initiatives are urgently needed if we are to stem emissions of forest carbon, and the loss of irreplaceable biodiversity, caused by rubber expansion onto forest in mainland Southeast Asia. These include: (1) a rubber sustainability initiative that restricts market access for deforestation rubber and/or offers a price premium for non-deforestation rubber; (2) zero-deforestation pledges from major corporate rubber consumers; and 3) governmental regulatory or economic incentives for forest conservation that couple improved forest governance with effective land-use planning. One emerging tool is the High Carbon Stock (HCS) approach, currently being considered for land-use planning of oil palm in Southeast Asia^54^, but it is currently unclear whether land covers classified as appropriate for agricultural conversion (vegetation cover with ≤75 per tC per ha or defined as “shrub and open lands”^54,55^) would encompass the open forests of mainland Southeast Asia. Should HCS gain widespread traction, an urgent policy priority is to ensure that open forests are protected under HCS. Finally, if such incentives or actions are appropriately designed to also tackle threats from other cash crops, such as cassava, this could unlock the potential for carbon markets to help protect the unique forests of mainland Southeast Asia from selective logging.

## Methods

### Study region

We used data from Cambodia as a case study for lowland areas of mainland Southeast Asia, and for the Indo-Burma biodiversity hotspot, that covers Laos, Cambodia, Vietnam, most of Myanmar and Thailand, and parts of Southwest China, including Xishuangbanna and Hainan Island^56^. Ten million ha of forest covered 55% of Cambodia in 2010^25^, but the country now has the world’s fifth highest deforestation rate^57^. Cambodian forests range from fully deciduous to almost completely evergreen^35,58^ but can be categorised into two broad groups: “dense forest” comprises evergreen and semi-evergreen stands, with tree heights reaching 35–55 m; “open forest” comprises areas of dry deciduous dipterocarp and mixed-deciduous forests, with tree heights reaching 25–35 m^22^. Swamp and hill evergreen forest types found in some periodically inundated or mountainous areas^35,58^ were not considered in this study.

The expansion of rubber is strongly promoted by the Cambodian government; rubber area increased by 175% between 2009 and 2013, to 328,800 ha^59^. Cashew, cassava and sugar areas have also increased: cassava by 163% from 2009, to 421,000 ha in 2013^59^; sugarcane by 76% from 2009, to 23,810 ha in 2013^59^; and cashew by 275% from 2000, to 60,000 ha in 2005, with no recent data available^60^. Timber is logged both illegally, and legally under licences for infrastructure projects and industrial-scale plantations (Economic Land Concessions), including of rubber^48,53,61,62^. Illegal logging of high-value timber is common, involving a range of actors^63^ (Supplementary Note 1). Smallholders seeking agricultural land for subsistence or cash crops, firewood and timber also drive deforestation^22^.

Opportunities for using forest carbon finance to protect forests in mainland Southeast Asia are being actively explored. Cambodia, alongside Vietnam and Lao PDR, is being supported by the UN-REDD (Reducing Emissions from Deforestation and Forest Degradation) program^64^, and has begun the REDD + Readiness process with assistance from the World Bank’s Forest Carbon Partnership Facility (FCPC) and UN-REDD, in anticipation of developing a national-level program^64^. A number of pilot REDD+ demonstration projects are underway, seeking funding from voluntary carbon markets^65^.

### Modelling opportunity costs and carbon breakeven prices

Opportunity costs were defined as forgone direct profits from logging, the net present value (NPV) of rubber in large plantations or of other cash-crop agriculture (cassava, cashew, and sugarcane, in large plantations or smallholdings), and cash income from collecting dipterocarp tree resin, which is forgone once dipterocarp trees are felled (Supplementary Note 2). This traditional livelihood activity directly conflicts with logging, because resin-producing species also have valuable timber^66^, being listed as class II species (Supplementary Data 1). Total opportunity cost was thus based on forgone profits from logging and/or rubber, offset by lost resin revenue where class I and II trees are logged out. We do not estimate other non-timber forest product benefits (Supplementary Note 2).

We did not distinguish between legal and illegal activities, as we wished to understand the underlying economic drivers of forest conversion, while acknowledging that when designing actual incentive mechanisms there may be good reasons for treating legal revenue streams differently from illegal ones, so as to avoid indirectly rewarding illegal behaviour. However, timber revenue was calculated as a farm-gate price and we did not consider legal downstream benefits that accrue to the wider economy, nor those benefits of doubtful legality that accrue mainly to non-local actors, including agro-industrial companies and elite logging “tycoons”^48,53,62^, such as the sale of high value timber on the international market (Supplementary Table 8, although export of logs and most sawn timber is illegal; Supplementary Note 1). These exclusions are likely to lead to an under-estimate of opportunity cost, but are appropriate given the absence of any robust data on these benefits, and the need for any forest carbon finance scheme to operate transparently and legally. Hence, our calculations provide a minimum estimate of the economic challenge that a forest carbon finance scheme may face in influencing land use decisions.

To calculate carbon breakeven prices, we incorporated the following parameters: timber profit (assuming a single offtake; $ per ha), forest carbon stock (tC per ha), post-deforestation land-use carbon stock (tC per ha), 25-year discounted resin revenue (10% discount rate; $ per ha) and 25-year NPV of rubber or cash crops (10% discount rate, $ per ha). All input costs and prices were adjusted to 2013 US$ before analysis; all output values are thus in 2013 US$.

When calculating each parameter, to account for both uncertainties within, and variance between, data sources, values were resampled for each of 10,000 model iterations from either a normal distribution defined by the mean and standard error of the mean (SE; used to resample timber volume, carbon stock values and agricultural farm gate prices, for which the distribution of values was known), or a uniform distribution bounded by minimum and maximum estimates (used to resample agricultural yields, agricultural input costs, timber prices and timber extraction costs, for which the underlying distribution of parameter values was unknown; Supplementary Table 9).

All parameter values were resampled independently at each iteration, with the exception of timber volume (m^3^ per ha) and carbon stock (tC per ha). For these, a single forest inventory was randomly selected for each model iteration, in order to capture geographic variation without bias from relative inventory extent (Supplementary Table 1), and thereby avoiding the need for weighting. For each forest inventory, the mean and SE of timber volume, carbon stock and stem density (Supplementary Data 1) were calculated per tree species, and thus each royalty class. Where trees of smaller size classes were sampled from subplots nested within main plots (Supplementary Table 1), standardised values (per ha) still allowed mean and SE to be calculated per royalty and size class and, as the numbers of subplots and main plots were equal within each such inventory (Supplementary Table 1), no weightings were required. From the selected inventory, timber volumes and carbon stocks were simultaneously sampled from the same point in their distribution relative to the mean, as values of timber volume and carbon stock were likely to be correlated.

Stem-specific timber volume and carbon stock estimates (derived separately from DBH) were negatively skewed across all forest inventories. To address this, timber and carbon densities per plot were square-root transformed before calculating the mean and SE for each forest inventory, reducing the influence of infrequent plots with exceptionally high timber and carbon density. Timber and carbon values resampled from the resulting normal distribution were back-transformed before use in the model. Carbon stocks and wood volumes are presented in results as the unweighted mean and 95% confidence interval across all 10,000 iterations.

Agricultural yields, costs and farm-gate prices for all crops were sampled independently for each iteration, but the position within the distribution for each parameter was held constant across each crop type for each iteration. Resin production and prices were also sampled independently for each iteration. By sampling yields, input costs and farm-gate prices independently, we underestimate variability in NPV relative to that potentially arising if variation in these parameters were correlated, for example due to site location. Similarly, although parameter values varied between model iterations, we assumed a constant temporal yield-curve over the 25 years and did not incorporate inter-annual stochastic variability.

Opportunity costs of logging and rubber (or other cash-crops; offset by lost resin revenue; $ per ha) and breakeven carbon prices needed to offset the opportunity costs, plus setup (transaction) and implementation costs (based on emitted forest carbon offset by post-deforestation land-use carbon stocks; $ per tCO_2_) were then generated for each scenario. Results are presented as the median and interquartile range across 10,000 model iterations. Indicative real-world carbon prices (Supplementary Table 4) are shown in relation to breakeven carbon prices.

### Timber profits

Forest inventories (Supplementary Fig. 2) were used to estimate timber volumes. Five inventories used fixed sampling areas (3.1–60 ha total per landscape; Supplementary Table 1) while inventory F05 used variable radius plots^67^. Within forest types, sites had similar relative distributions of size classes for all trees (Supplementary Fig. 4, Supplementary Table 10) and within royalty classes (Supplementary Fig. 9). In each, all trees ≥ 10 cm DBH were measured and identified to species level; nomenclature was standardised across datasets following The Plant List Version 1.1 (2013)^68^. Vines were not recorded. Tree species were assigned to one of five royalty (value) classes: luxury, I, II, III, or non-classified (NC; Supplementary Data 1) based on a government list of timber species^69^. The 14 luxury timber species included Burmese Rosewood (*Dalbergia oliveri*, commonly called *D. bariensis* in Cambodia (Supplementary Data 1), IUCN EN, CITES Appendix II), Siamese Rosewood (*D. cochinchinensis*, VU, CITES Appendix II) and Burmese Padauk (*Pterocarpus macrocarpus*, unassessed). All *Dipterocarp* species were class I or II and included popular timber species (e.g. *Dipterocarpus alatus*, EN and *Anisoptera scaphula*, CR); other popular timber species in class I were *Sindora siamensis*, LC, and *Heritiera* (*Tarrietia*) *javanica*, unassessed^63^).

Logging revenues depend on the minimum tree size commercially harvested, which may differ according to royalty class. Luxury species are exceptionally valuable and even small amounts are harvested^70^; we therefore assumed all luxury trees ≥ 10 cm DBH would be logged. For class I and II species, we assumed a minimum harvestable DBH of 40 cm, but also explored the effect of reducing minimum harvestable DBH to 30 cm (Supplementary Note 1; Supplementary Table 2). Class III trees (that are used for local construction or fuelwood) and non-classified timbers (assumed to be only useful as fuelwood) ≥ 40 cm DBH were initially assumed to have market value (Supplementary Table 8). However, extraction of class III and non-classified timbers was found to incur a net cost in open forest (−$57, −$456 to −$21 per ha) and in dense forest (−$1,336, −$2,096 to −$850 per ha), despite assuming relatively low timber extraction costs, that involved selective logging activity by local people in Cambodia in a “business-as-usual” scenario with no formal logging concessions, inventories, management plan, or demarcation of logging areas. Costs included: wage labour, food, motorbike fuel, ox-cart transportation to the roadside/village and chainsaw maintenance, but excluded the capital cost of the chainsaw. If class III and non-classified classes were to be extracted, this would likely be for firewood or construction locally (with extraction costs subsumed within non-market subsistence livelihood activities), or through destructive clearance during land preparation for agriculture (already considered in establishment costs for scenarios of agricultural conversion; Supplementary Table 11). Therefore, maximum potential timber profit accrued solely from logging luxury, class I and class II timber, and we assumed that the opportunity cost of protecting forests from further logging after the “Luxury, I, II timber logged” scenarios was reduced to zero (Fig. 3, Supplementary Table 2).

The timber profit remaining in the forest (*R*_*x*_; $ per ha) in each logging scenario *x*, representing the opportunity cost of intervening at that point to protect timber remaining within the forest from further logging, was calculated as shown in equation (1):

1 $$R_x = \mathop {\sum}\limits_{{{a}} = 1}^5 {V_a \times \left( {p_a - C} \right)}$$

Where *V*_*a*_ is the timber volume (m^3^ per ha) of timber royalty class *a* (five classes; Supplementary Data 1), estimated using Cambodian government standard timber equations (Supplementary Table 12) reduced by 20% to account for wastage^71^, *p*_*a*_ is the timber price ($ per m^3^) for that royalty class (Supplementary Tables 8 and 13), and *C* the cost ($ per m^3^) of extraction to the roadside or village (Supplementary Table 14). Timber prices were estimated at the local (roadside or village) level in the absence of formal timber markets.

### Forest carbon stocks

As for timber volume, forest inventories (Supplementary Table 1) were used to estimate forest carbon stocks for all stems ≥ 10 cm DBH, per royalty class, per forest plot, quantifying AGB and BGB^22^. AGB per plot and per royalty class (t per ha) was calculated using the Chave D Moist forest equation, using DBH only^72^, verified via destructive sampling for a REDD + pilot project in Cambodia^22^. Species-specific wood density was used where possible^73^; other species were assigned the mean wood density for the genus within the same region or, if no values were available for the genus, the mean wood density of all tree species across all inventories. BGB was estimated as 24% of AGB per plot^74^. AGB and BGB were summed, and multiplied by 0.5 to give estimated carbon content (tC per ha)^72^. Deadwood and SOC pools were not estimated; deadwood accounts for only 3% of emissions reductions from avoided deforestation, and SOC stocks are assumed not to change when land use conversion is to perennial crops according to the Intergovernmental Panel on Climate Change (IPCC) tier 1 carbon outcome calculation methods, and the carbon accounting methodology used for the REDD + pilot project in Cambodia^22^.

### Post-deforestation land-use carbon stocks

We estimated the AGB and BGB carbon stocks of post-deforestation land-use classes which may partially offset forest carbon emissions. Time-averaged carbon stocks (taCs) were estimated for each crop type, which give the mean C stock over a plantation cycle from planting to harvesting^3,75^. This approach allows carbon stock estimates to be scaled up from a single plot to the landscape level, comparison of land uses with different rotation lengths, accommodates clearance and carbon release at the end of the crop rotation, and better reflects the net carbon outcomes and long term climate impact of a transition from one steady-state land use to another than a time series of carbon fluxes^76,77^. The taCs approach is consistent with the IPCC Good Practice Guidelines^78^ and the carbon accounting methodology used for the REDD + pilot project in Cambodia^22^.

The carbon stock estimate for rubber (52.5 tC per ha) was based on multiple estimates of taCs calculated either as the carbon stock in the median year of the plantation cycle using logistic or Gompertz models of growth, or 50% of the carbon stock in the final year of the plantation cycle assuming a linear biomass increase^3^. Time-averaged carbon stocks of other crops (cashew 22.3 tC per ha, sugarcane 6.8 tC per ha, cassava 2.5 tC per ha) were estimated as 50% of the carbon stock accumulated at the maximum rotation length (Supplementary Table 6). As for forests, soil carbon stocks were not considered, although there is strong evidence for soil carbon reductions when forest is converted to rubber^3^ or other tree cash crops^27^.

### Resin revenue

The potential revenue generated by resin collection over a 25-year period (*D;* $ per ha; years 0–24 inclusive) was calculated following equation (2):

2 $$D = \mathop {\sum}\limits_{{{n}} = 0}^{24} {\frac{{\left[ {\left( {t \times i} \right) \times y} \right] \times p_R}}{{\left( {1 + r} \right)^n}}}$$

where *t* is resin tree stem density per ha (from forest inventories), *i* the likely proportion of productive trees (i.e., excluding non-starter or exhausted trees that do not yield), *y* the resin yield (litres per stem per year) and *p*_*R*_ the resin price ($ per litre; Supplementary Table 15). Resin revenue is discounted over a 25-year timeframe *n* (years 0–24 inclusive) using a discount rate *r* of 10%, with no discount applied in year 0. All trees ≥ 30 cm DBH can potentially be tapped, with no identified relationship between resin yield and DBH; labour costs were not included in calculation of resin profits, as resin tends to be collected only when there are few or no alternative wage options^66^.

### Agricultural net present value

We estimated likely farm-gate profits for rubber and other cash crops using region-specific data (Supplementary Tables 5 and 11). The 25-year discounted net present value (NPV, $ per ha; *P*_*b*_) of each potential crop (*b*; rubber, cashew, cassava, sugar) was calculated as shown in equation (3):

3 $$P_b = \mathop {\sum}\limits_{{{n}} = 0}^{24} {\frac{{\left( {y_b \times p_b} \right) - C_b}}{{\left( {1 + r} \right)^n}}}$$

where *y*_*b*_ is the year-specific yield (t per ha per year), *p*_*b*_ the price ($ per t) and *C*_*b*_ the cost of production ($ per ha per year). Profits are discounted over a 25-year timeframe *n* (years 0–24 inclusive) using a discount rate *r* of 10%, with no discount applied in year 0. In the case of annual crops, an “end-of-year” perspective is taken, whereby returns accrue in the same year as crop establishment.

A comparison of the spatial distribution of historically suitable environmental space for rubber^1^ and the spatial distribution of deciduous dipterocarp forest (DDF^24^; which shares many characteristics with our open forest category, although often in fine-grained mosaic with mixed deciduous and other forest types) shows most DDF lies outside the optimal zones for rubber cultivation. Reduced rubber yields were predicted (though the magnitude of reduction was not defined) in areas of drought risk, defined as <60 mm rainfall per month for >5 months per year, and/or <1,200 mm rainfall per year and/or <20 mm rainfall during the driest quarter^1^. This drought risk definition overlaps with the bioclimatic limits of DDF (1,000–1,500 mm rainfall per year with a defined dry season)^24^. Although rubber yield reductions due to drought have not been quantified, reduced dry season growth can delay the onset of tapping from the sixth to the tenth year after planting^36^. We therefore delayed the onset of tapping in the plantation cycle in open forest scenarios (Supplementary Table 11).

To accommodate change in annual yield across a 25-year production cycle, model iteration-specific yield curves were simulated separately for each crop (Supplementary Fig. 10, Supplementary Table 11), using a single iteration-specific randomly-generated yield index (proportionate between minimum and maximum values, uniform distribution). Crop-specific production costs were sampled from a uniform distribution between the minimum and maximum values available in the literature (Supplementary Table 11) and crop-specific farm-gate prices were used (Supplementary Table 5 and 11).

### Breakeven carbon prices

The breakeven carbon price (*E*_*xb*_; $ per tCO_2_) required to offset the opportunity cost of forest conservation ((*R*_*x*_*–D*) + *P*_*b*_) and cover the costs of REDD + project setup (one-off transaction cost) and implementation (discounted over 25 years; *G*; $ per ha) for each scenario of logging (*x*) and crop (*b*, including the option of no crop) was calculated as shown in equation (4):

4 $$E_{xb} = \frac{{\left[ {\left( {R_x - D} \right) + P_b} \right] + G}}{{3.67 \times Z_x}}$$

where *Z*_*x*_ is the residual carbon stock (tC per ha) of all trees ≥ 10 cm DBH remaining in each logging scenario (*x*) and 3.67 the conversion factor from tC to tCO_2_^28^. Carbon stocks of post-deforestation land-uses were subtracted from *Z*_*x*_ when exploring the impact of incorporating these stocks on carbon breakeven prices. Estimated PES project setup ($4.95 per ha) and implementation costs ($9.47–$13.09 per ha per year) were obtained from a multi-year spending history and projected management expenditure budget for a pilot REDD + project in Cambodia (Wildlife Conservation Society, unpublished data). These costs fell well within annual management and implementation cost estimates in existing literature, that range from $0.87–$20.01 per ha^79^. Annual implementation costs were discounted and summed across a 25-year timescale.

Finally, sensitivity analyses explored the impacts on carbon breakeven price of: increasing or decreasing timber, resin and agricultural commodity prices (Supplementary Fig. 6), non-availability of resin markets (Supplementary Table 7), reducing the threshold of commercially viable stem diameter on timber profits (Supplementary Table 2), and alternative discount rates of 5%, 8 and 15% as applied to agricultural NPV and resin revenue (Supplementary Figs. 7, 8).

### Leakage

The costs of controlling for leakage of avoided deforestation for rubber, or forest degradation through selective logging, were not included in analyses. Ultimately, the need for land for rubber expansion will only be mitigated through reduction in global demand for natural rubber, which is reliant upon 1) global markets and demand for products such as vehicle and aircraft tyres, 2) development of alternatives to natural rubber, or 3) improvements in recycling methods. Similarly, demand for timber, within and beyond mainland Southeast Asia, would need to be met from well-managed sources before leakage of forest degradation or conversion could be effectively controlled. However, a robust rubber sustainability initiative, or corporate zero deforestation commitments, may displace rubber plantations to sites where land use conversion entails negligible net carbon emissions. We have therefore not attempted to incorporate the cost of controlling leakage in this analysis.

### Data availability

A summary of the agricultural data used for this study are shown in Supplementary Table 11, with data sources detailed in Supplementary Table 5. Original forest inventory data (Supplementary Table 1, Supplementary Fig. 2) are not publicly available, and were made available for sole use in this study with permission of the following organisations and co-authors: F01 by Permian Global in collaboration with Ecometrica and Birdlife Cambodia; F02 and F03 by the Forestry Administration of the Royal Government of Cambodia, Wildlife Conservation Society Cambodia Program and Wildlife Conservation Society Global Conservation Program; F04 by Cambodia Development Resource Institute (CDRI); F05 by D.P. Bebber and F.H. Lambrick; and F06 by I. Theilade, J.F. Maxwell and P. Chhang. Derived data supporting the findings of this study, and all R scripts used to resample data and run the models, are available from the corresponding author on reasonable request.

## Electronic supplementary material

Supplementary Information

Description of Additional Supplementary Files

Supplementary Data 1
