# Supplementary material for: Protecting tropical forests from the rapid expansion of rubber using carbon payments
Source: Nat Commun. 2018 Mar 2;9:911. doi: 10.1038/s41467-018-03287-9 (PMC5834519; doi:10.1038/s41467-018-03287-9)
Supplement: Supplementary file 1 — Supplementary Information [file 41467_2018_3287_MOESM1_ESM.pdf]

Supplementary Figure 1

Producer price comparison among data sources. Panels show comparison of producer prices from different data sources for: a) rubber, b) sugar, c) cassava and d) cashew. Prices shown are nominal dollars adjusted to US$ 2013 dollars using a consumer price index (CPI) averaged across Thailand, Vietnam and Cambodia.

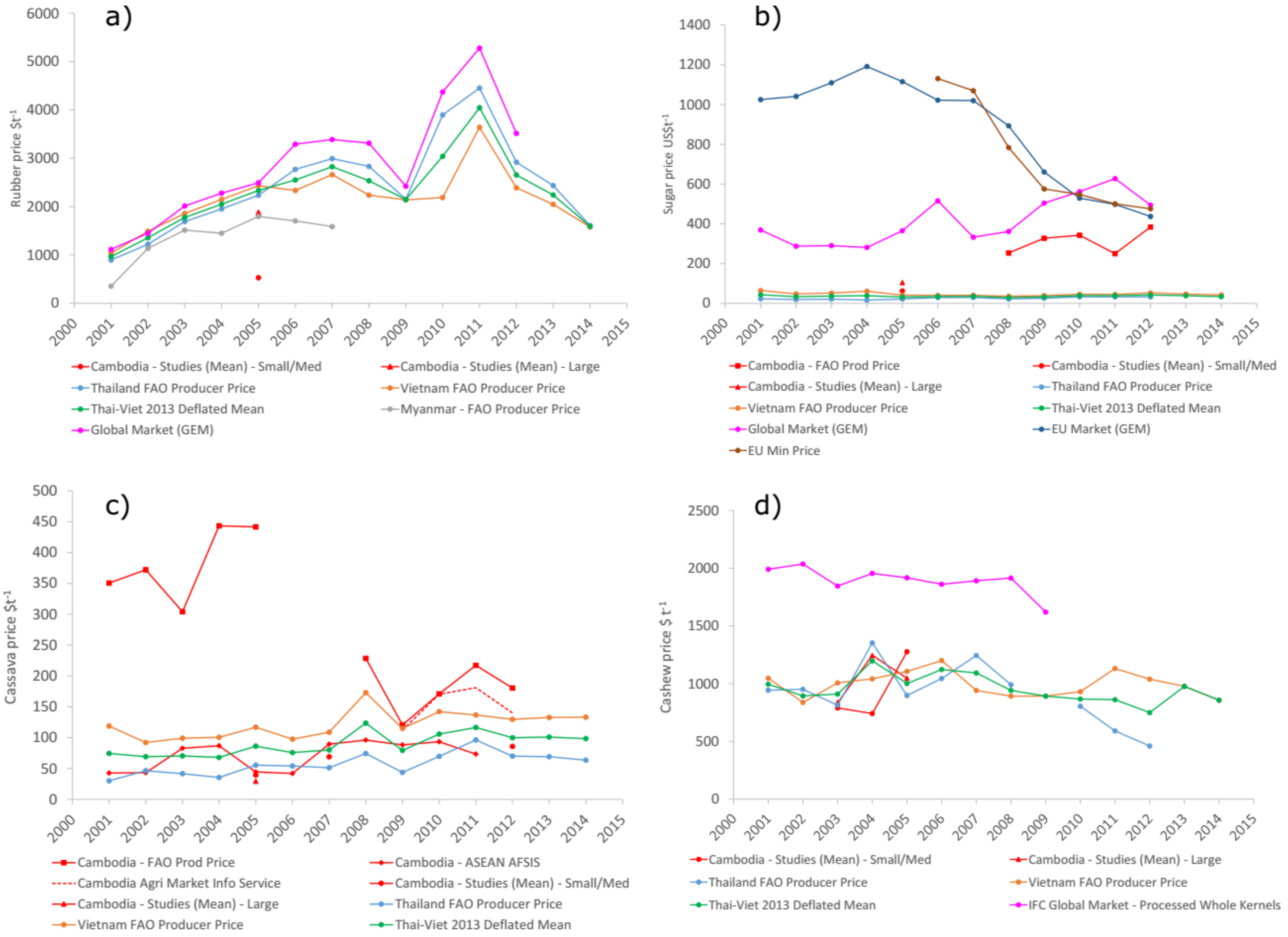

Supplementary Figure 2

Map of forest inventory locations. Forest inventories were obtained from six landscapes (F01 – F06) in Cambodia (Supplementary Table 1); dense forest was sampled in each landscape, and open forest in three landscapes. Two landscapes are managed for biodiversity conservation (F01 and F02), two are partly managed by communities (F04 and F05) and two are not under formal management (F03 and F06). Some selective logging had taken place in all landscapes prior to data collection, as has occurred across most of the region (Supplementary Note 1). F03 is represented by a single marker as the inventory comprised a single 60 ha plot, and F04 is represented by a single marker as individual plot locations were not available. Dense forest was sampled at all locations, and open forest at F01, F02 and F04. Data sources: F01 (Permian Global in collaboration with Ecometrica and Birdlife Cambodia, unpublished data, 2009); F02 & F03 (Wildlife Conservation Society/Forestry Administration, unpublished data, 2011); F04 (CDRI, unpublished data, 2006[1]); F05[2]; F06[3] (with the late J.F. Maxwell). Forest cover, protected areas and water bodies extent obtained from Open Development Cambodia (https://opendevelopmentcambodia.net), licensed under CC-BY-SA (https://creativecommons.org/licenses/by-sa/3.0).

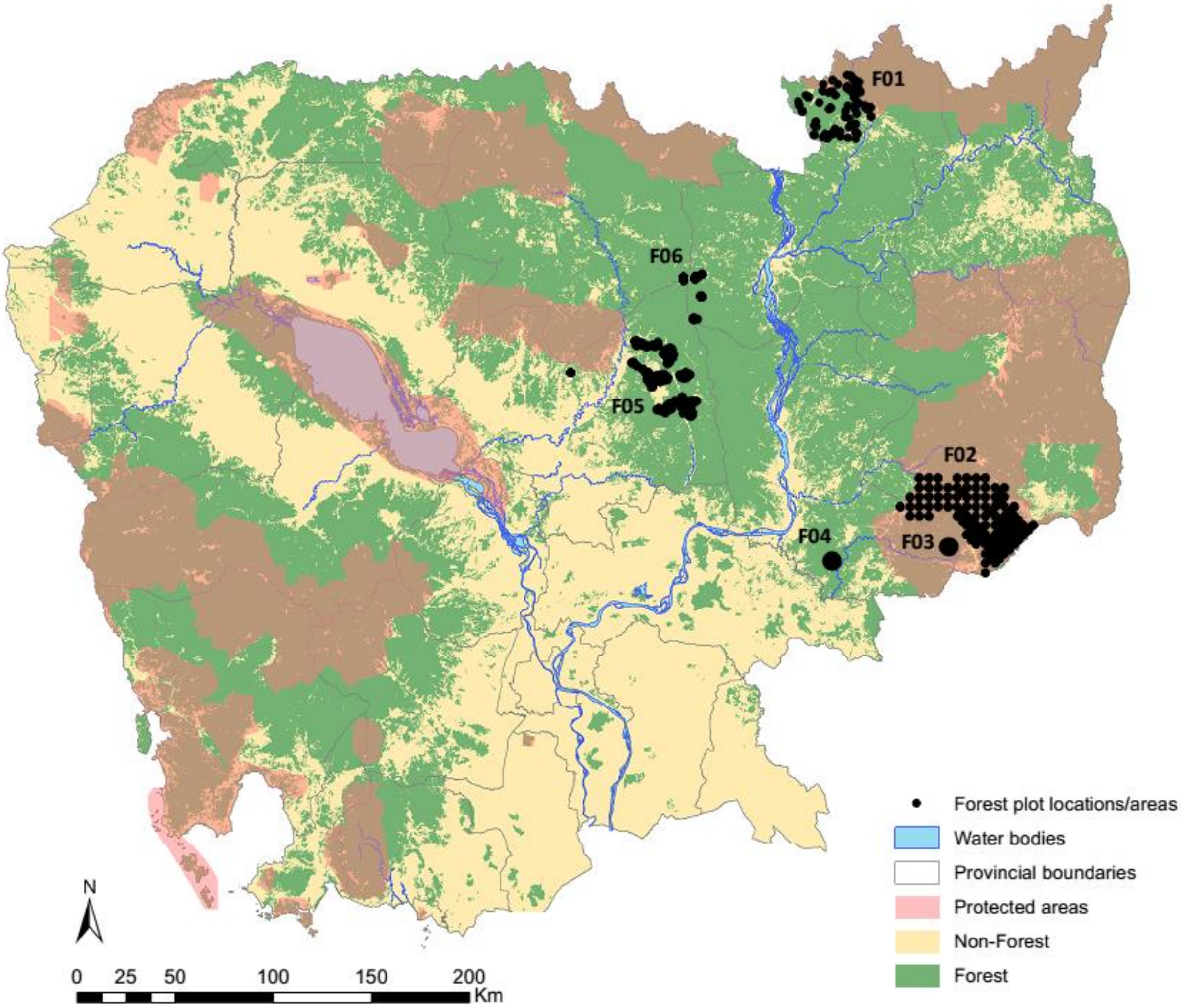

Supplementary Figure 3

Forest carbon stock of dense and open forest in each of six Cambodian forest landscapes. The between-landscape variation in carbon stock (tC ha^{-1}), shown for a) dense forest and b) open forest, reflects differences in annual rainfall, dry season length, and species composition and structure[4,5], but may also reflect exploitation history.

a

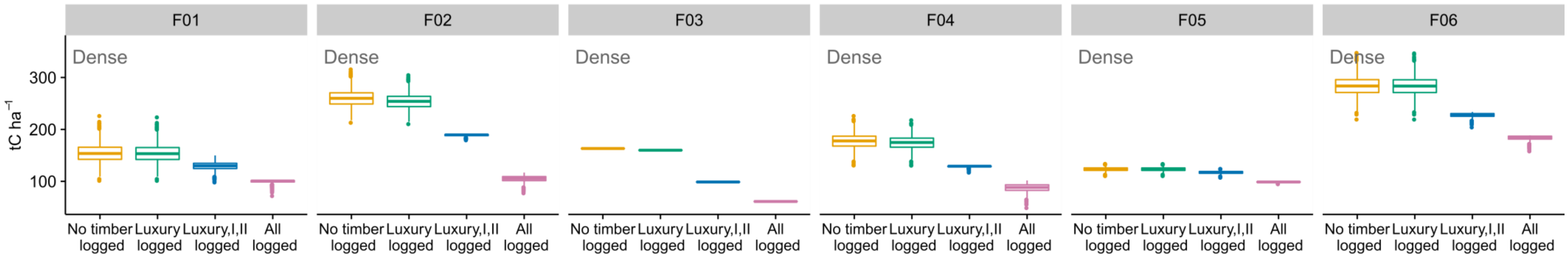

b

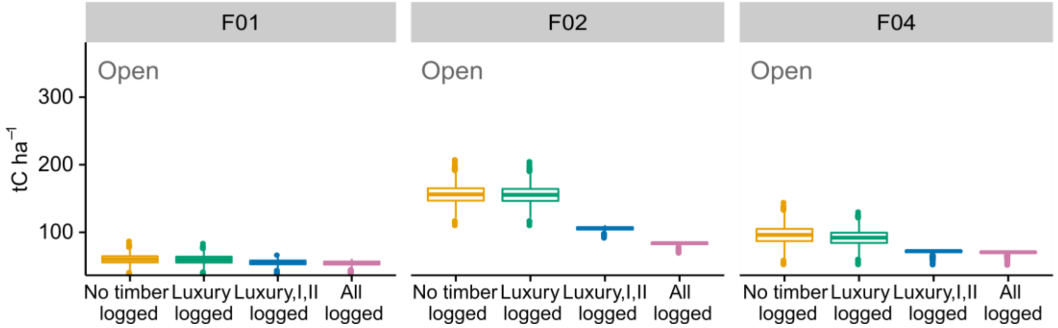

Supplementary Figure 4

Frequency distribution of stem diameters for each forest inventory. Data only includes stems ≥30cm in a) dense forest and b) open forest. Bars show the relative frequency of trees within DBH size categories, while curves represent a smoothed density distribution. Frequency distribution of tree sizes is similar across dense forest landscapes, while in open forest landscapes. Although open forest in landscapes F02 and F01, and dense forest in landscape F02 appeared to have a greater proportion of larger luxury class stems (≥60cm DBH) than other landscapes, the differences were not significant (Supplementary Figure 9; Supplementary Table 11).

a) Dense forest

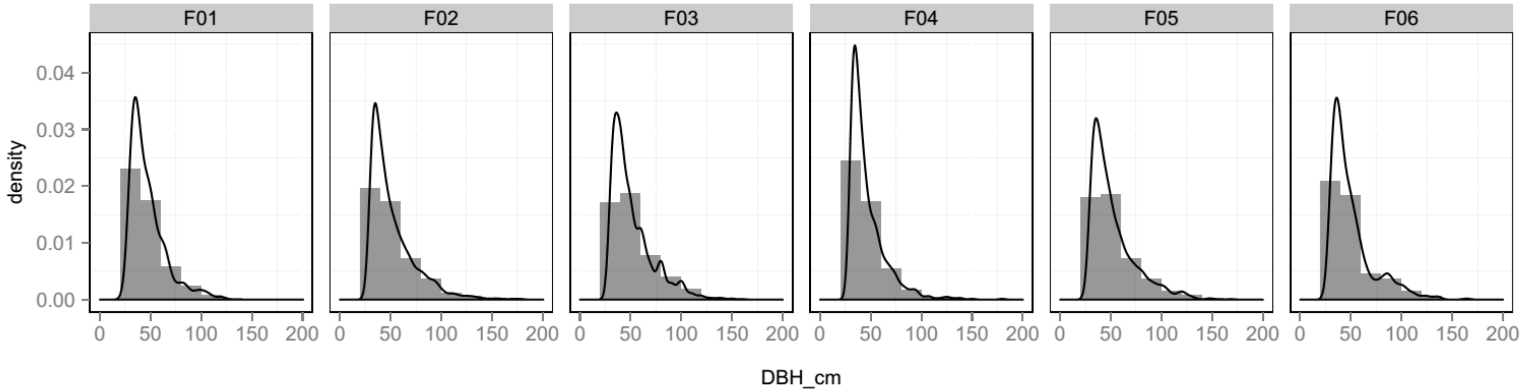

b) Open forest

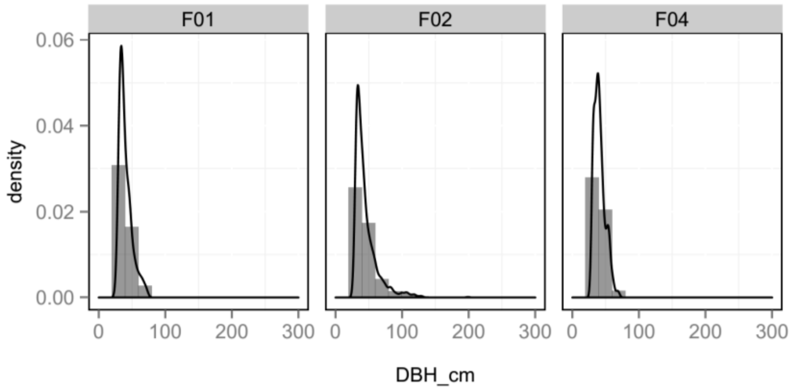

Supplementary Figure 5

Opportunity costs and breakeven carbon prices needed to protect forests from logging and conversion to cassava, cashew and sugar. Opportunity costs (a – f) are based on the forgone profits from logging (for luxury class timber, trees ≥10 cm DBH, other classes of timber, trees ≥40 cm DBH) and agriculture (cassava, cashew or sugar, 25 year NPV, 10% discount rate), offset by lost revenue from resin collection with the logging of resin trees (timber royalty class II), which are logged in the "Luxury, I, II logged" and "All logged" scenarios. Breakeven carbon prices (g – l) are the prices needed to offset opportunity costs, REDD+ setup costs and implementation costs. Costs are shown separately for dense and open forests. Time-averaged post-deforestation land use carbon stocks partially offset forest carbon losses. Grey lines in the BCP panels represent real world carbon prices (Supplementary Table 10): dotted = \$5 tCO_2^{-1} (indicative of voluntary market forest carbon sales and non-market carbon fund prices), short dash = \$13 tCO_2^{-1} (indicative of compliance market prices) and long dash = \$36 tCO_2^{-1} (indicative of the social cost of carbon). Outliers (more than 1.5x the interquartile range) are not displayed to improve the clarity of the figure; the value shown above each box-whisker gives the *n* outliers excluded out of 10,000 modelled results.

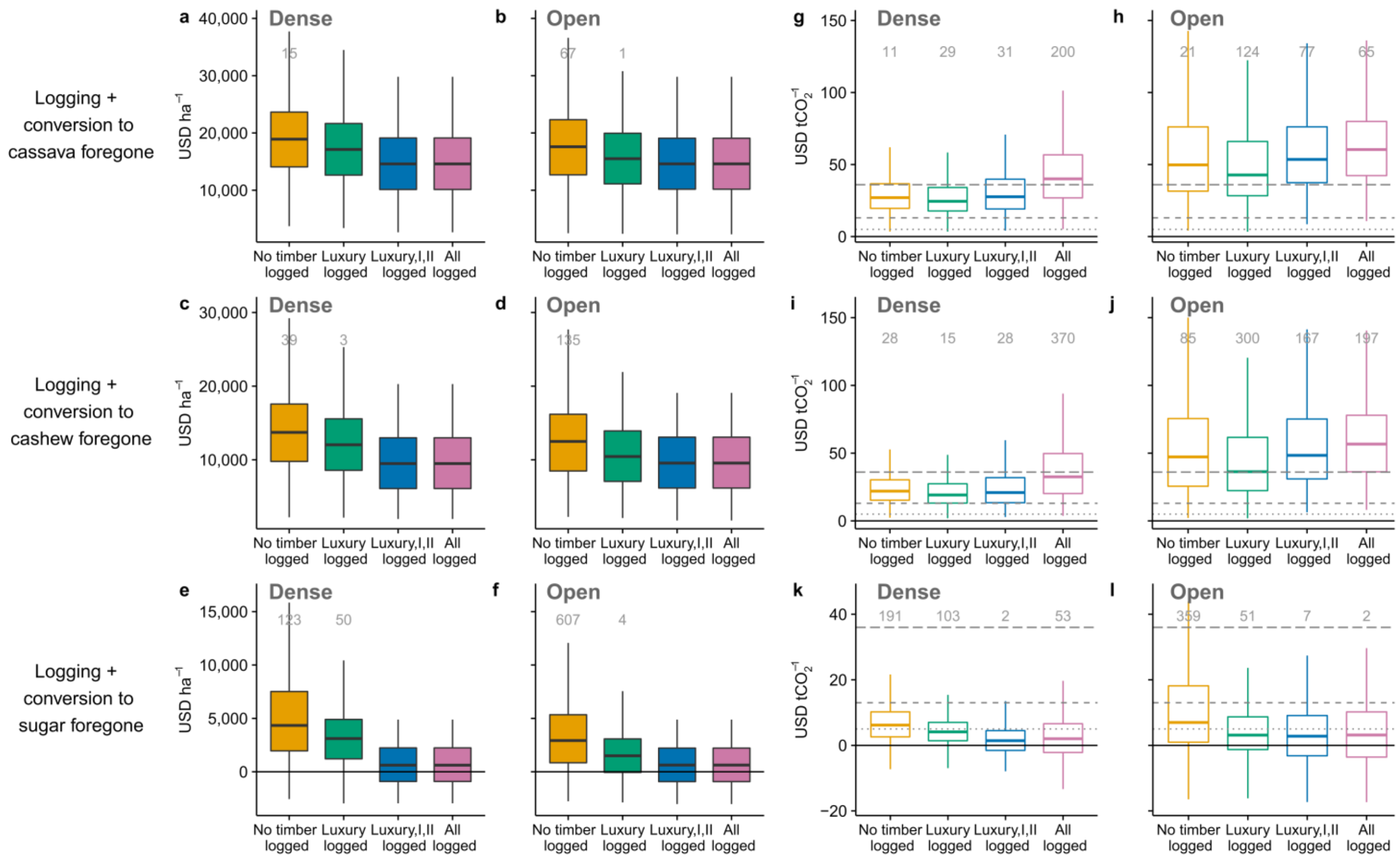

Supplementary Figure 6

Sensitivity analysis assessing the effect of commodity prices on breakeven carbon prices. Sensitivity analyses explored the impacts of increasing and decreasing a) timber, b) agricultural and c) resin prices on carbon breakeven price, using a price index randomly drawn from a uniform distribution between 0.3 and 2 (i.e. from less than a third, to two times the mean price), following Gilroy et al[6]. This index range encompassed all annual producer farm gate price fluctuations for natural rubber 2000 – 2014 inclusive. For each input variable examined (timber, agricultural or resin price), carbon equilibrium prices are shown in relation to the price index. For agricultural price sensitivity analysis, we only show sensitivity of carbon breakeven prices under the "No timber logged" scenario for each crop. Increases in timber and agricultural prices could have a strong effect on the opportunity costs of conservation. In contrast, resin prices would need to increase by more than two times their current value in order to reduce carbon equilibrium prices. Grey lines on the panels represent real world carbon prices (Supplementary Table 10): dotted = \$5 tCO_2^{-1} (indicative of voluntary market forest carbon sales and non-market carbon fund prices), short dash = \$13 tCO_2^{-1} (indicative of compliance market prices) and long dash = \$36 tCO_2^{-1} (indicative of the social cost of carbon).

a) Timber prices – "Logging only"

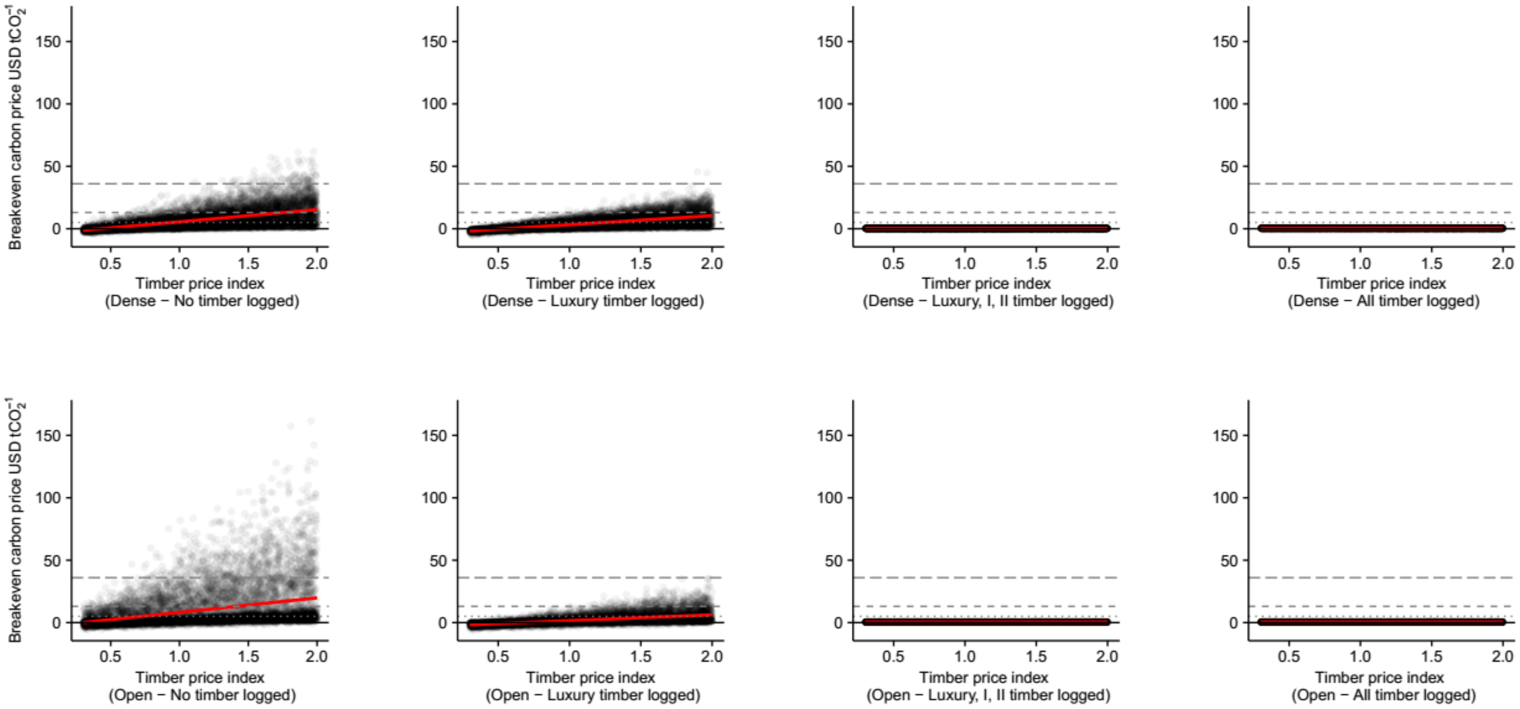

b) Agricultural prices – "No timber logged"

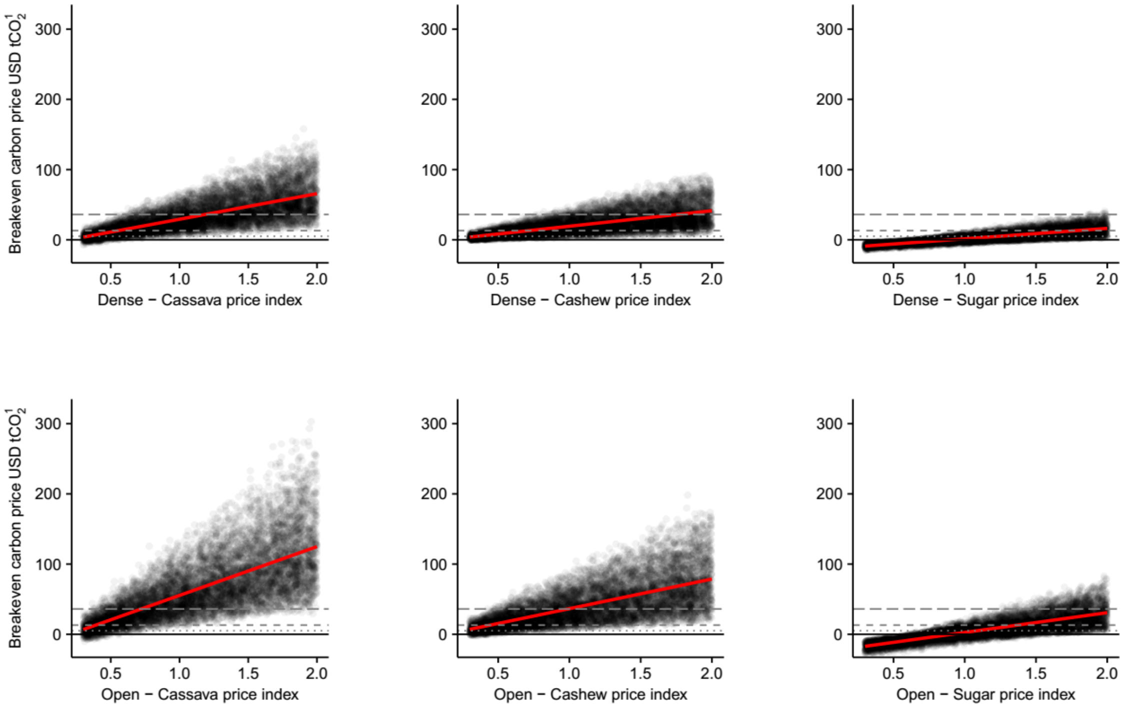

c) Resin prices - “Logging only”

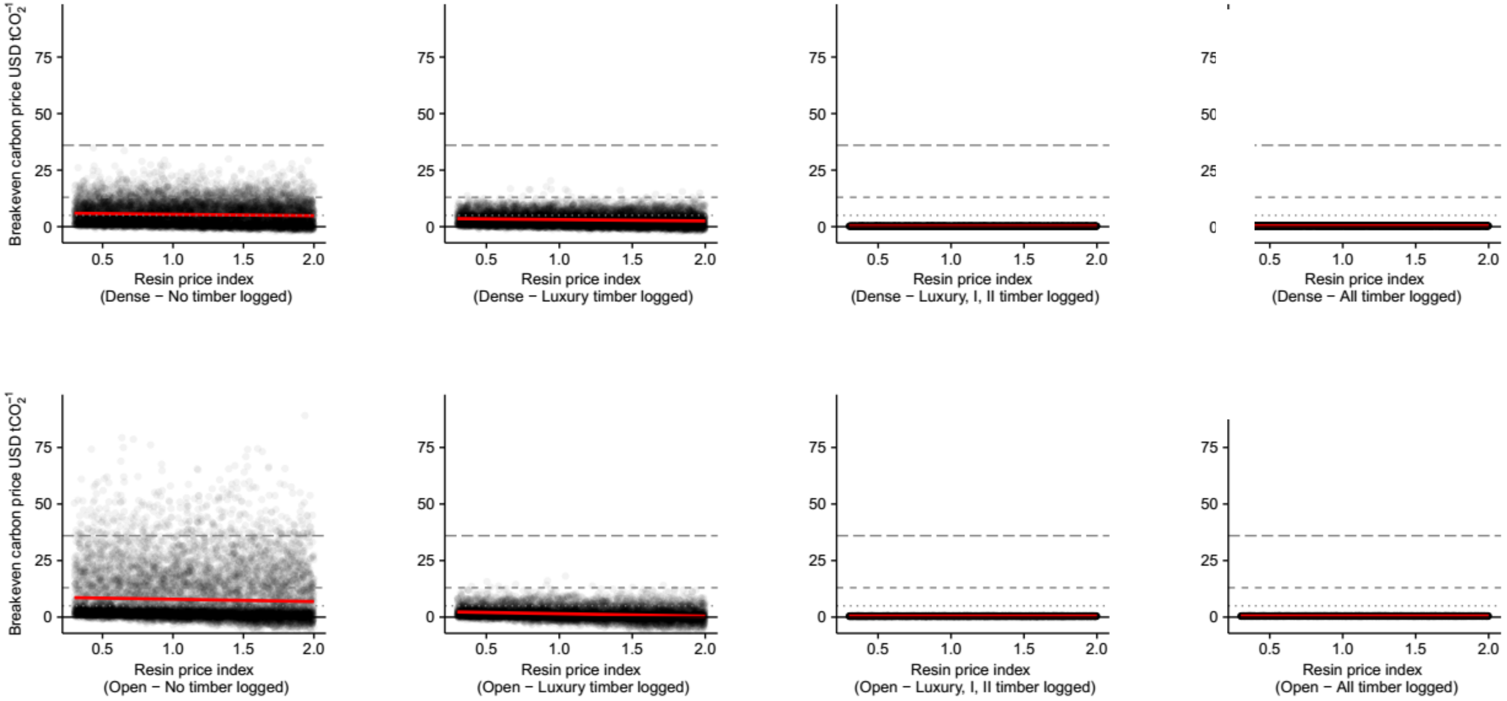

Supplementary Figure 7

Consequence of an alternative 5% discount rate on opportunity costs and breakeven carbon prices needed to protect forests from logging and conversion to rubber and other cash crops. Opportunity costs (OC; a – j) include forgone profits from logging (for luxury class timber, trees ≥10 cm DBH, other classes of timber, trees ≥40 cm DBH), and/or conversion to agriculture, offset by resin revenue, except where resin trees (class II) are logged out (in the "luxury, I, II logged" and "all timber logged" scenarios). Breakeven carbon prices (BCP; k – t) are the prices needed to offset opportunity costs, REDD+ setup costs and implementation costs. Costs are shown separately for dense and open forests. Time-averaged post-deforestation land use carbon stocks partially offset forest carbon losses. Grey lines on BCP panels represent real world carbon prices: dotted = \$5 tCO_2^{-1} (indicative of voluntary market forest carbon sales and non-market carbon fund prices), short dash = \$13 tCO_2^{-1} (indicative of compliance market prices) and long dash = \$36 tCO_2^{-1} (indicative of the social cost of carbon). Outliers (more than 1.5x the interquartile range) are not displayed to improve the clarity of the figure; the value shown above each box-whisker gives the *n* outliers excluded out of 10,000 modelled results.

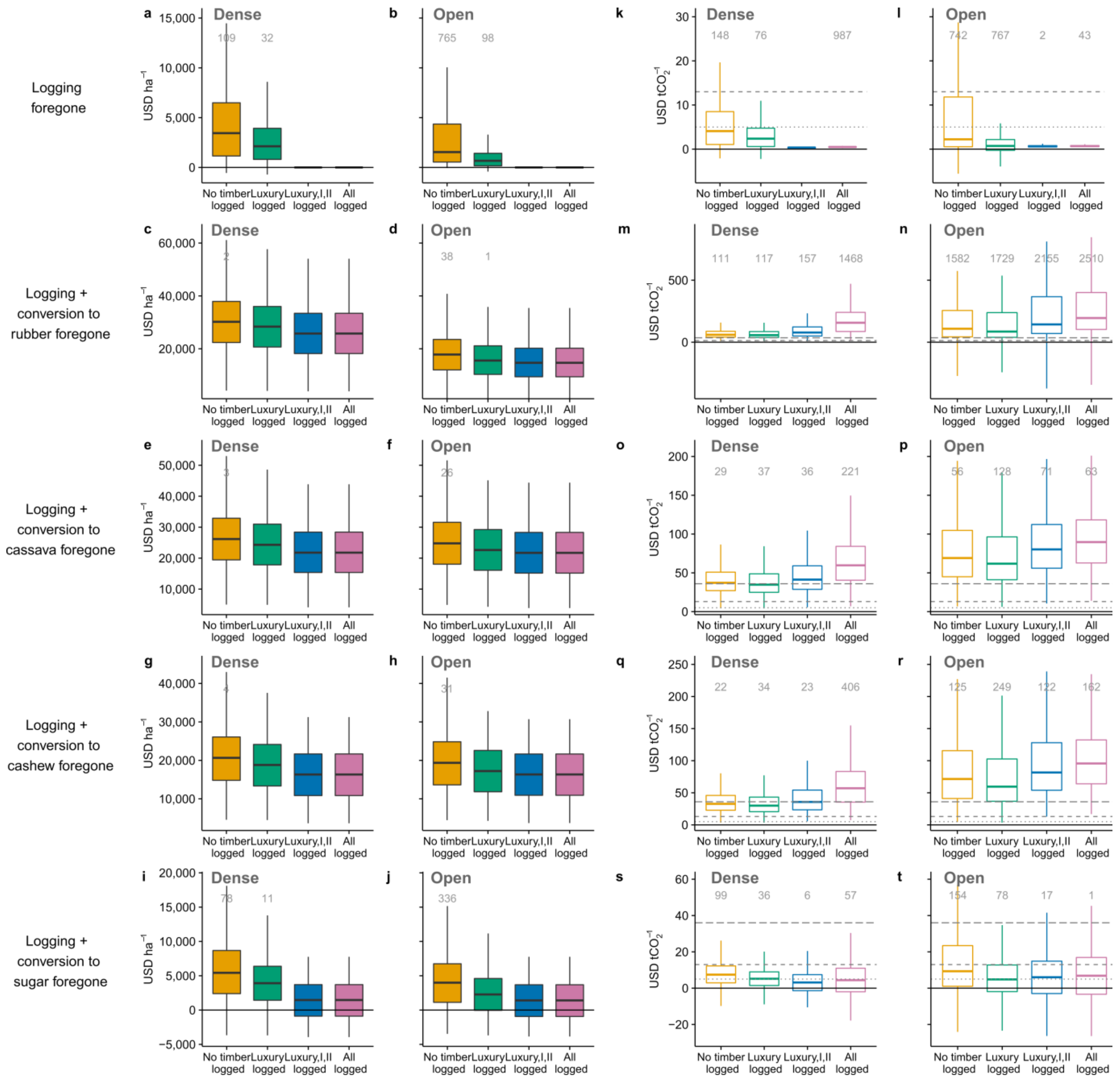

Supplementary Figure 8

Consequence of an alternative 15% discount rate on opportunity costs and breakeven carbon prices needed to protect forests from logging and conversion to rubber and other cash crops. Opportunity costs (OC; a – j) include forgone profits from logging (for luxury class timber, trees ≥10 cm DBH, other classes of timber, trees ≥40 cm DBH), and/or conversion to agriculture, offset by resin revenue, except where resin trees (class II) are logged out (in the “luxury, I, II logged” and “all timber logged” scenarios). Breakeven carbon prices (BCP; k – t) are the prices needed to offset opportunity costs, REDD+ setup costs and implementation costs. Costs are shown separately for dense and open forests. Time-averaged post-deforestation land use carbon stocks partially offset forest carbon losses. Grey lines on BCP panels represent real world carbon prices: dotted = \$5 tCO_2^{-1} (indicative of voluntary market forest carbon sales and non-market carbon fund prices), short dash = \$13 tCO_2^{-1} (indicative of compliance market prices) and long dash = \$36 tCO_2^{-1} (indicative of the social cost of carbon). Outliers (more than 1.5x the interquartile range) are not displayed to improve the clarity of the figure; the value shown above each box-whisker gives the *n* outliers excluded out of 10,000 modelled results.

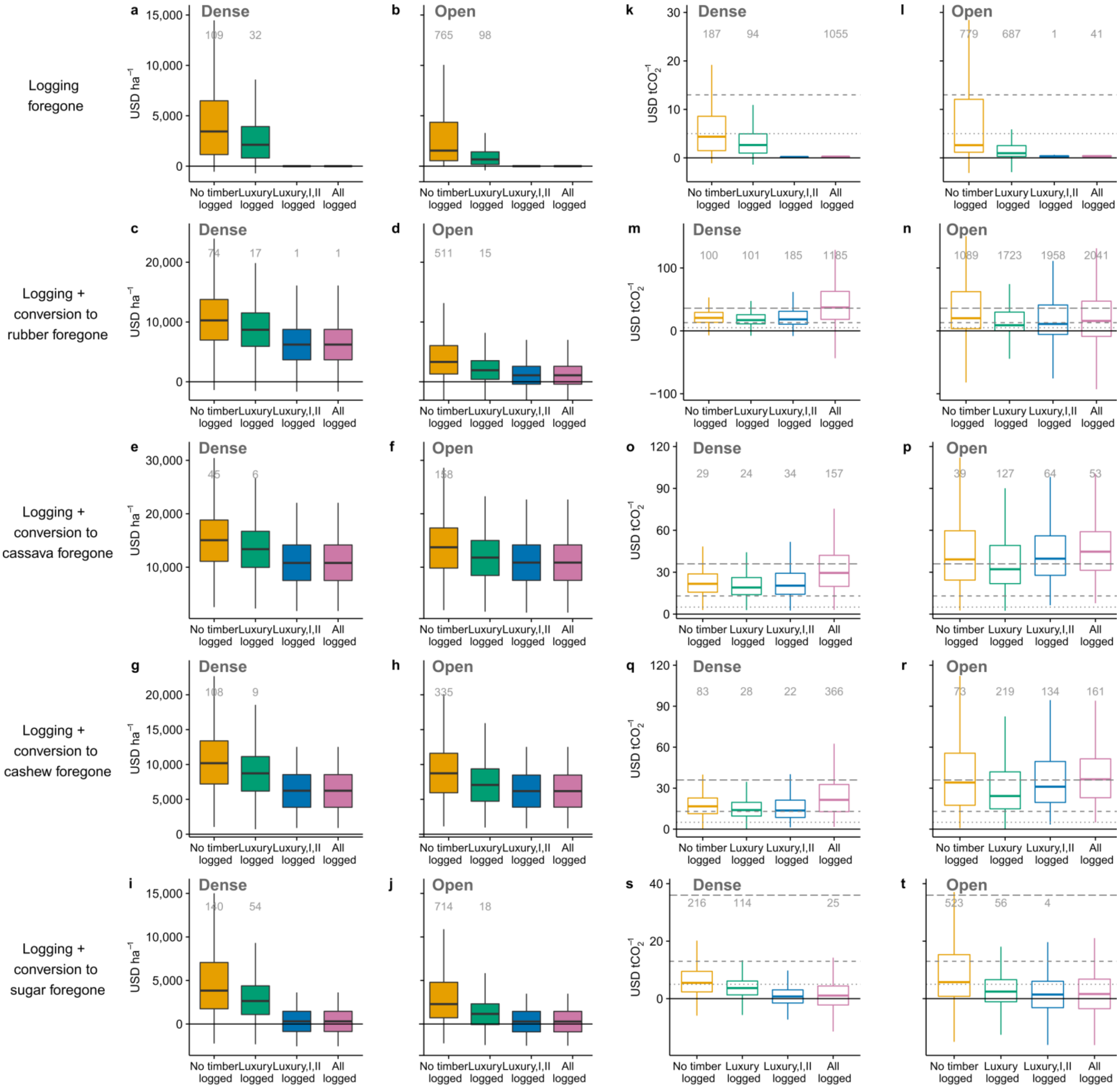

Supplementary Figure 9

Frequency distribution of stem diameters for each timber royalty class within each forest inventory. Data only includes stems ≥30cm in dense forest (a, i – vi) and open forest (b, i – iii). Bars show the relative frequency of trees within DBH size categories, while curves represent a smoothed density distribution. Frequency distribution of tree sizes is remarkably similar across all forest landscapes.

a) Dense forest

i. F01

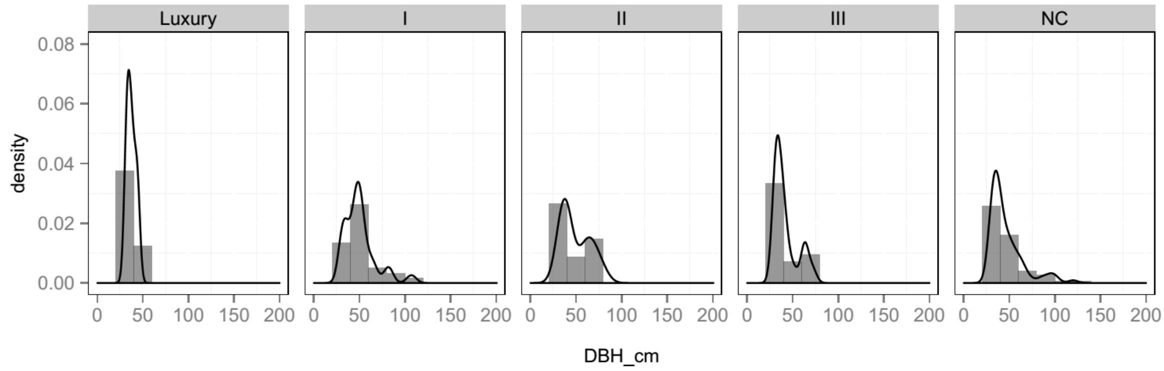

ii. F02

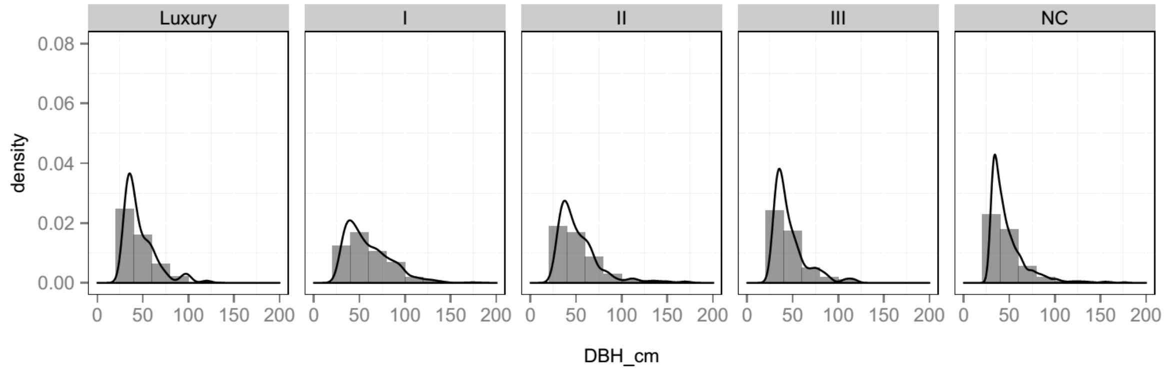

iii. F03

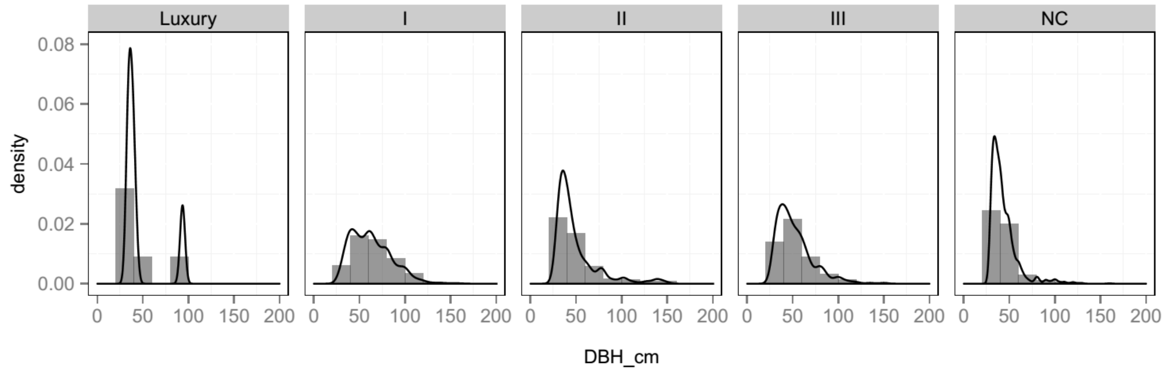

iv. F04

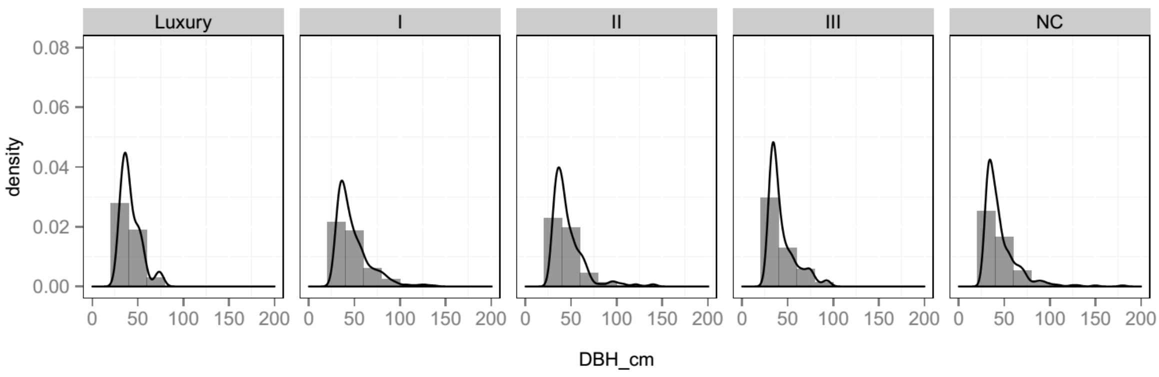

v. F05

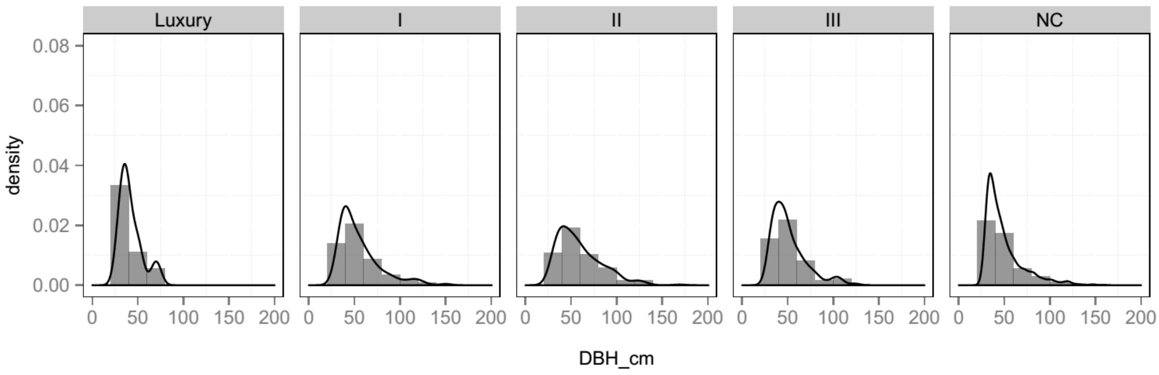

vi. F06

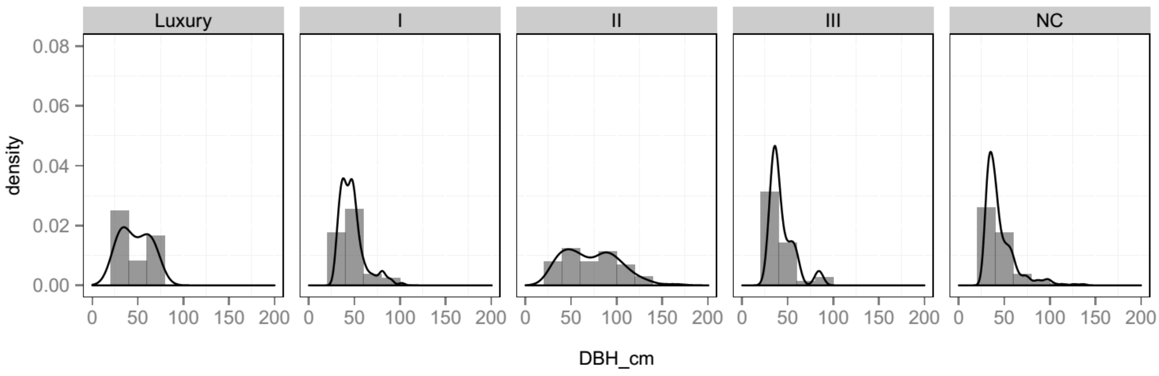

b) Open forest

i. F01

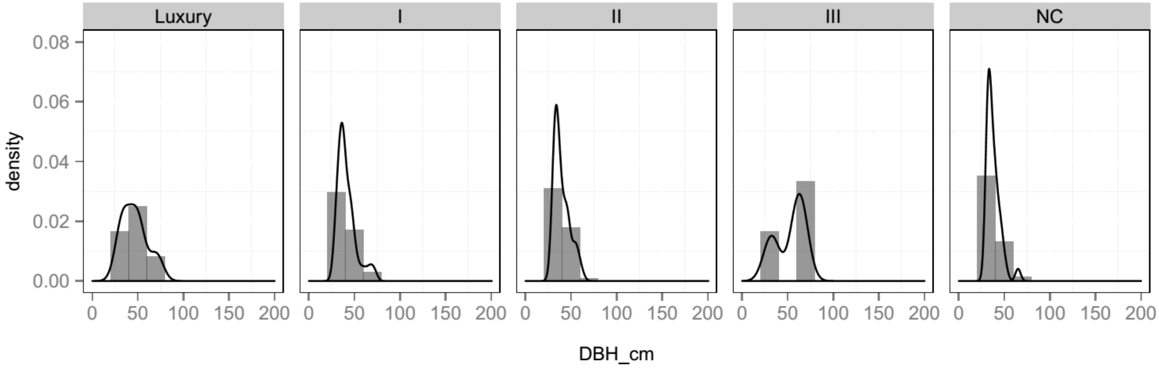

ii. F02

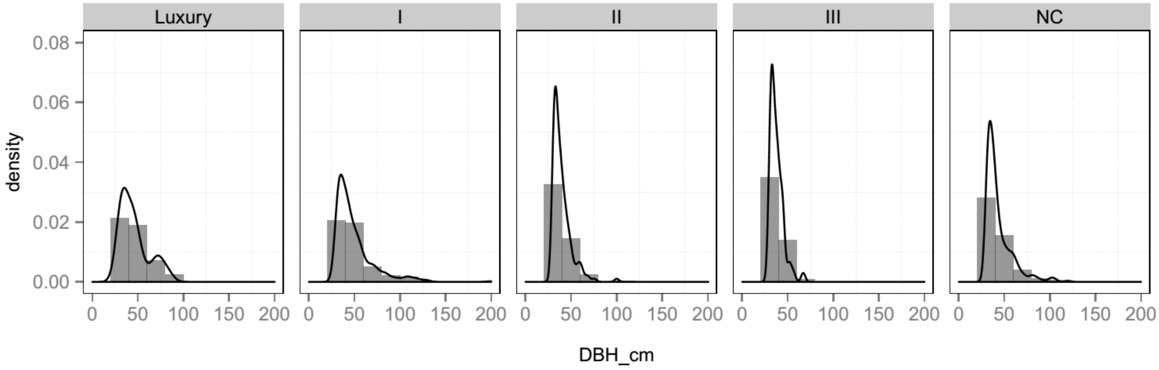

iii. F04

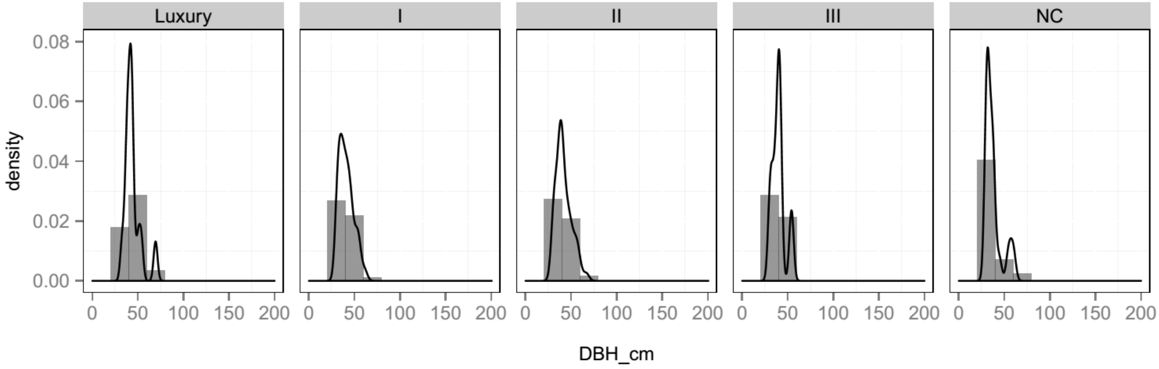

Supplementary Figure 10

Rubber yield curve simulation example. For explanation of simulation, see Supplementary Table 5.

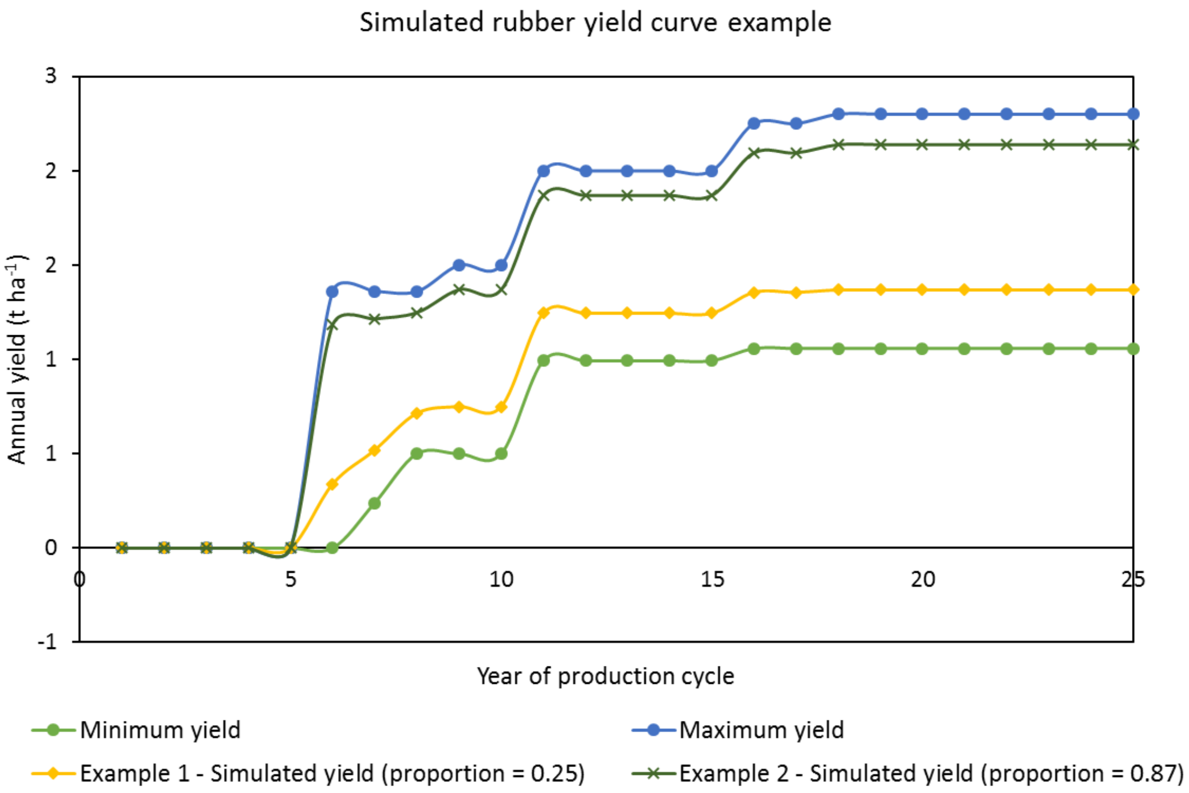

Supplementary Table 1

Tree inventories used to parameterise carbon and timber models. Inventories were originally collected for forest structure, carbon stock measurement and forest composition studies. Five inventories used a fixed sampling area (3.1 - 60 ha total per landscape) while F05 used variable radius plots. Where data were available for trees 5 ≤ 10cm DBH, these were omitted from analysis, standardising to a minimum DBH of 10cm across all datasets. Data sources: F01 (Permian Global in collaboration with Ecometrica and Birdlife Cambodia), F02 and F03 (Wildlife Conservation Society/Forestry Administration, unpublished data, 2011), F04 (CDRI, unpublished data, 2006[1]), F05[2], F06[3] (with the late J.F. Maxwell).

Forest category	Source	Forest type	n trees >10 cm DBH	n trees >30 cm DBH	Tree size (DBH, cm)	n plots	Plot dimensions (m)	Plot area (ha)	Total area sampled (ha)	Approx. coverage (max. distance between plots, km)
Dense	F01	Semi-Evergreen	457	188	5 - 20	31	Radius = 5.64	0.01	0.31	38
					>20	31	Radius = 17.84	0.1	3.10	
	F02	Evergreen/Semi-Evergreen	4594	2289	5 – 15	63	3x subplots; radius = 5	0.024	1.48	60
					15 – 30	63	3x subplots; radius = 15	0.212	13.36	
					>30	63	3x subplots; radius = 20	0.377	23.75	
	F03	Evergreen	3246	2784	5 - 15	24 (pooled to 1)	10 X 25	0.025	0.60	n/a
					15 - 30	24 (pooled to 1)	10 X 50	0.05	1.20	
					>30	1	1000 x 600	60	60.00	
	F04	Evergreen/Semi-Evergreen	3038	894	5 – 10	24	25 x 50	0.125	3.00	n/a
					10 – 30	24	50 x 50	0.25	6.00	
					>30	24	50 x 100	0.5	12.00	
	F05	Evergreen, Semi-Evergreen + Deciduous	3013	1349	>10	361	n/a - variable radius	n/a	n/a	48
	F06	Evergreen, Riverine, Lagostroemia	2512	576	>10	100	10 x 50	0.05	5	83
**Total**			16860	8080		585			129.80	260
Open	F01	Deciduous	373	128	5 – 20	36	Radius = 5.64	0.01	0.36	29
					>20	36	Radius = 17.84	0.1	3.60	
	F02	Deciduous/Semi-Evergreen matrix	2366	1193	5 – 15	41	Radius = 5	0.024	0.97	60
					15 - 30	41	Radius = 15	0.212	8.69	
					>30	41	Radius = 20	0.377	15.46	
	F04	Deciduous	682	195	5 – 10	15	25 x 25	0.0625	0.94	n/a
					10 – 30	15	25 x 50	0.125	1.88	
					>30	15	50 x 50	0.25	3.75	
Total			3421	1516		92			35.65	260
	**Grand Total**		**20281**	**9596**		**667**			**165.45**	**260**

Supplementary Table 2

Median and interquartile range of timber profits for each scenario.

	**Opportunity cost of protection (median, \$ ha^{-1})**	**Opportunity cost of protection (lower 25% quartile, \$ ha^{-1})**	**Opportunity cost of protection (upper 25% quartile, \$ ha^{-1})**	**% timber value removed**	**Remaining % timber profit**
Minimum harvestable DBH: 10cm for luxury, other classes ***40cm***					
**Dense**					
No timber logged	3,443	1,151	6,490	0	100.0
Luxury timber logged	2,123	810	3,931	38.3	61.7
Luxury, I, II timber logged	0	-	-	100.0	0
All timber logged	0	-	-	100.0	0
**Open**					
No timber logged	1,543	543	4,356	0	100.0
Luxury timber logged	671	165	1,422	56.5	43.4
Luxury, I, II timber logged	0	-	-	100.0	0
All timber logged	0	-	-	100.0	0
Minimum harvestable DBH: 10cm for luxury, other classes ***30cm***					
**Dense**					
No timber logged	4,308	1,637	7,537	0	100.0
Luxury timber logged	2,881	1,227	4,967	33.1	66.9
Luxury, I, II timber logged	0	0	0	100.0	0
All timber logged	0	0	0	100.0	0
**Open**					
No timber logged	3,373	1,214	6,346	0	100.0
Luxury timber logged	1,961	710	3,494	41.9	58.1
Luxury, I, II timber logged	0	0	0	100.0	0
All timber logged	0	0	0	100.0	0

Supplementary Table 3

Median and interquartile range of agricultural 25-year net present value (NPV), with input costs and prices in $US adjusted to 2013. NPV is shown for large, monocultural plantations of rubber and sugar, and smallholder farms of cashew and cassava

Forest type	Discount rate (%)	Crop type	25-year NPV (median, $ ha^{-1})	Lower 25% quartile ($ ha^{-1})	Upper 25% quartile ($ ha^{-1})
Dense	5	Rubber	25,774	18,191	33,423
		Cassava	21,782	15,373	28,414
		Cashew	16,328	10,847	21,680
		Sugar	1,463	-886	3,707
	8	Rubber	16,533	11,403	21,733
		Cassava	16,980	12,000	22,041
		Cashew	11,762	7,490	15,838
		Sugar	919	-856	2,784
	10	Rubber	12,571	8,436	16,698
		Cassava	14,597	10,133	19,124
		Cashew	9,491	6,099	12,989
		Sugar	619	-905	2,238
	15	Rubber	6,224	3,676	8,763
		Cassava	10,800	7,511	14,167
		Cashew	6,236	3,862	8,549
		Sugar	306	-860	1,461
Open	5	Rubber	14,697	9,414	20,219
	8		7,909	4,463	11,464
	10		5,089	2,532	7,764
	15		1,088	-403	2,591

Supplementary Table 4

Real world carbon prices. Indicative carbon prices were sought from the literature for comparison with estimated breakeven carbon prices. Three prices were selected, chosen to represent voluntary markets and carbon funds, compliance markets, and the estimated social cost of carbon, respectively.

Mean price ($\$ tCO_2^{-1}$)	Price type	Source	Indicative price used ($\$ tCO_2^{-1}$)
1.70	All forest carbon offsets sold on the voluntary market in 2014	7	5.00
3.70	Avoided deforestation (REDD) credits sold globally in 2014	7	
5.00	Non-market forest carbon payments (e.g. bilateral agreements between Norway/Guyana)	7	
5.40	All voluntary market carbon sales	7	
12.70	All compliance market carbon sales (e.g. California's cap and trade policy)	7	13.00
18.00	Corporate internal carbon prices	8	
36.00	US government social cost of carbon	9	36.00
40.00	World Bank social cost of carbon	8	

Supplementary Table 5

Data sources for agricultural net present value calculation. For each parameter, price and cost estimates from different sources were adjusted to US$ 2013 using an averaged CPI for Thailand, Cambodia and Vietnam[10] before calculation of means, variance or range. FAO producer prices are compared to price estimates from other sources, including GEM commodity prices, in Supplementary Figure 1. For each parameter an explanation of data treatment and value selection is given; resulting crop-specific parameter values are provided in Supplementary Table 8.

Parameter	Reference	Country	Method
Rubber – large scale monocultural plantation crop			
**Rubber producer price**<br><br>**(see also Supp. Fig. 1)**	[11]	Thailand/ Vietnam	Producer prices are not published for Cambodia on FAOSTAT[11], so the mean and standard error of the mean (SE) of annual producer prices across Thailand and Vietnam (2003 – 2012 inclusive) were used to account for price volatility and regional variation in rubber prices. Compared to prices reported from ground surveys in Cambodia ($530 – 1878 t^{-1}; data from 2005 adjusted to US$ 2013; [1,12–14] these values ($1355 - $4046, data from 2002 – 2012, $2333 in 2005) are relatively high; farmers, especially smallholders, may receive lower prices in Cambodia. The most recent data from 2014 gives a producer price of $1,644 t^{-1}. We do not include returns from felling the rubber trees and selling the timber at the end of the 25 year cycle as this is often used to cover the costs of replanting the next cycle [15].
**Rubber yield**	[1,12,13]	Cambodia	Yield data for each production year of the 25-year management cycle of a large plantation were extracted from each study. Minimum and maximum yields for each year, across all studies, were used as minimum and maximum input parameters for the simulation model (n = 3). Yield estimates that appeared to use unclear units which could correspond to wet latex yield rather than dry rubber yield, were excluded from analysis. For each model iteration an annual dry rubber yield curve (t ha^{-1} yr^{-1}) over 25 years was generated as a proportion of the maximum attainable yield. For each iteration (i), a proportion ($prop_i$ range 0 to 1) was selected randomly from a uniform distribution. Then for each production year (t, range 0-24) the run-specific yield ($Y_{i,t}$) was calculated applying this proportion to the interval between reported minimum ($Ymin_t$) and maximum ($Ymax_t$) yields, following the equation: $Y_{i,t} = Ymin_t + (Ymax_t\text{-}ymin_t) * Yprop_i$. The run-specific proportion used to create the yield curve was generated independently for each crop, for each iteration. Two examples, for $prop_i$ = 0.25 and 0.87, are shown in Supplementary Figure 10. For open forests, a yield penalty was applied to account for predicted slower tree growth in dry conditions by delaying the onset of tapping until 10 years after planting, rather than the usual 6 years[16].
**Rubber production cost**	[1,12–14]	Cambodia	Production cost data for each year of the 25-year management cycle for large monocultural plantations were extracted from each study (n = 2 referring to large plantations), standardised to USD ha^{-1} yr^{-1} in US$ 2013. Costs included: land clearance (using bulldozer or tractor, year 1 only, n = 1), land preparation (annual), planting material, annual inputs, annual labour costs. We do not include the costs of felling rubber trees at the end of the 25-year management cycle as this cost is associated with replanting and the start of the next yield production cycle.<br><br>For each model iteration an annual cost curve ($ ha^{-1} yr^{-1}) over 25 years was generated as a proportion of the maximum cost. For each iteration (i), a proportion ($Cprop_i$ range 0 to 1) was selected randomly from a uniform distribution. Then for each production year (t, range 1-25) the run-specific cost (C_{it}) was calculated applying this proportion to the interval between reported minimum ($Cmin_t$) and maximum ($Cmax_t$) costs, following the equation: $C_{i,t} = Cmin_t + (Cmax_t\text{-}Cmin_t) * Cprop_i$. The run-specific proportion used to create the cost curve was generated independently for each crop, for each iteration.

Cashew – smallholder monocultural plantation crop			
**Cashew producer price**<br><br>**(see also Supp. Fig. 1)**	[11]	Thailand/ Vietnam	Producer prices are not published for Cambodia on FAOSTAT[11], so the mean and standard error of the mean (SE) of annual producer prices across Thailand and Vietnam for 2003 - 2012 inclusive ($861 - $1197, data from 2002 - 2012, US$ 2013) were used to account for price volatility and regional variation in cashew prices Compared to prices reported from ground surveys in Cambodia ($789 - $1047, 2003 – 2005, in US$ 2013[1,12,13]), Cambodian farm gate prices are similar to producer prices in Thailand and Vietnam. Farmers are therefore likely to receive prices similar to these in Cambodia.
**Cashew yield**	[12]	Cambodia	Six sets of yield data, for different management cycle lengths, were extracted from ACI (2005[12]) for small farms, covering four provinces. Some yields seemed surprisingly high (up to 5.5 t ha^{-1}), given that average yields from a large scale plantation in Cambodia were reported to be 0.8 t ha^{-1} [1], and a report on the cashew industry in Cambodia states that the maximum known yields from Cambodia are 2.0 t ha^{-1}, with exceptional yields of 2.5 t ha^{-1} reported from Vietnam [17]. We therefore excluded any datasets reporting average yields >2.5 t ha^{-1} over a 25-year plantation cycle.<br><br>Yield curves reported for management cycles of less than 25 years (e.g. 10 or 15 years) were extrapolated according to the proportional yield declines reported for 25-year management cycles. Minimum and maximum yields for each production year, across all six yield curves, were used as minimum and maximum input parameters for the simulation model and an iteration-specific yield curve was simulated, as for rubber. The run-specific proportion used to create yield curve was generated independently for each crop, for each iteration.
**Cashew production cost**	[12]	Cambodia	Production cost data for each year of the 25-year management cycle were extracted from each of the six datasets in ACI (2005[12]), standardised to USD ha^{-1} yr^{-1} (adjusted to 2013). Costs included: land clearance (using manual labour, year 1 only), land preparation (year 1 only), planting material, annual inputs and labour costs (including imputed family labour costs). Where management cycles reported were for less than 25 years, costs were extended to year 25 based on final year costs in each dataset. Variation in reported labour requirements among the six datasets was high (total annual labour days ranged from 25 to 267 days ha^{-1}). On inspection of the budgets, this was accounted for by a ten-fold greater number of annual labour days reported for harvesting in one smallholder budget (220 – 300 days ha^{-1}). In three other smallholder datasets, annual harvesting labour days ranged from 16 – 30 days ha^{-1}, while a maximum of 35 days ha^{-1} were reported from a survey of 140 smallholder cashew farmers in Ghana. We therefore considered this large estimate of labour input to be an error, and limited the maximum annual harvesting days to 35 days ha^{-1}.<br><br>Minimum and maximum costs for each production year, across all six datasets, were used as minimum and maximum input parameters for the simulation model, and an iteration-specific cost curve was simulated, as for rubber. The run-specific proportion used to create the cost curve was generated independently for each crop, for each iteration.
Cassava – smallholder monocultural annual crop			
**Cassava producer price**<br><br>**(see also**	[11]	Thailand/ Vietnam	Producer prices for raw cassava are published for Cambodia on FAOSTAT[11], but they are much higher than any of the following price estimates for raw cassava, and therefore appear to be incorrect: producer prices for Thailand and Vietnam; farm gate prices for Cambodia reported in ACI[12] or Hing & Thun[14]; farm gate prices provided by The Cambodian Ministry of Agriculture, Forestry and Fisheries (MAFF) to

**Supp. Figure 1)**			the ASEAN Food Security Information System[18]; or local prices reported from up to 11 local markets across Cambodia ([19]; see Supplementary Figure 1).<br><br>Farm gate prices provided by MAFF were not available for all years of interest[18], so we used the mean and standard error of the mean (SE) of annual producer prices across Thailand and Vietnam for years 2003 – 2012, adjusted to US$ 2013 ($90.51 t^{-1}). This estimate is within the range of farm gate prices reported by MAFF to ASEAN ($42 – 96 t^{-1} 2001 – 2011, in US$ 2013[18], and prices reported by farmers in Cambodia in 2013 (n = 15, $75.00 – $90.00 t^{-1};[20])<br><br>As for rubber, we suggest that small farmers may receive lower prices where access to markets is limited. There is also variation in price among provinces depending on the degree of harvesting, drying and transportation costs paid by the farmer versus middlemen; we base our NPV estimates on the most commonly used system in Cambodia, in which farmers sell raw cassava to traders, and traders pay harvesting, drying and transport costs [14]
**Cassava yield**	12,14,21	Cambodia	Annual yield data (raw cassava, t ha^{-1} yr^{-1}) were extracted from each dataset. ACI[12] provided a number of estimates for different farm sizes, while Hing & Thun[14] and Sopheap et al[22] reported yields for smallholder farms only. Minimum (10.5 t ha^{-1} yr^{-1}) and maximum (30.0 t ha^{-1} yr^{-1}) yields were extracted for each year across all datasets, specific to smallholder farms. The mean of these values (23.8 t ha^{-1}) is close to the mean yield reported at the national scale in Cambodia (20.49 t ha^{-1} in 2007[22]), suggesting that these bounds are appropriate. Yields were assumed to remain constant over 25 years of repeated annual planting. This assumption is supported by data from Cambodia, that show no difference in yield between plots continuously cropped with cassava for 25 years without fertilisation and soils that had been cropped for <10 years[21]. Supporting this, yields of 13.3 t ha^{-1} yr^{-1} are reported from fully exhausted soils in Colombia[23]. However, cassava is potassium limited, and potassium-depleted soils can produce yields as low as 5 t ha^{-1} yr^{-1} in India, but if soils contain potassium-producing minerals, depletion does not occur even in absence of fertiliser application[23]**.** Cassava is tolerant to low soil fertility conditions[21,23,24]. Evidence from Cambodia suggests that soil type does not affect cassava yield, although farmers self-identified areas of higher soil quality where cassava yields were increased within individual farms[21]. We thus do not modify our yield estimates for open and dense forest areas, despite predicting that soil types may differ under each forest type. Yield curves were created for 25 consecutive years of cultivation, as for rubber and cashew, using the same proportion of maximum yield for each year. The run-specific proportion used to create yield curve was generated independently for each crop, for each iteration.
**Cassava production cost**	12,14	Cambodia	Annual production cost data for raw cassava were extracted from each of six datasets and standardised to USD ha^{-1} yr^{-1} units, adjusted to US$ 2013. Costs in all studies included: land clearance (using manual labour, year 1 only), land preparation (annual), planting material, annual inputs, annual labour costs (including imputed family labour costs), but did not include drying or transportation costs. Cost curves were created for 25 consecutive years, as for rubber and cashew, using the same proportion of maximum cost for each year. The run-specific proportion used to create the cost curve was generated independently for each crop, for each iteration.
Sugar – large scale monocultural annual crop			
**Sugar producer price**<br><br>**(see also Supp. Figure 1)**	11	Thailand / Vietnam	Producer prices are published for Cambodia on FAOSTAT[11], but are much higher than FAOSTAT prices for Thailand and Vietnam and much higher also than Cambodian farm gate prices reported in ACI[12]. Prices were checked against global prices, and EU minimum prices, as Cambodia receives preferential pricing to export sugar to the EU through the "Everything but Arms Treaty"[25]. However, Cambodia's Producer Prices were substantially lower than either of these indicators. We therefore used the mean and standard error of the mean (SE) of annual producer prices across Thailand and Vietnam for years 2003 – 2012 ($34.34 t^{-1}), but as for rubber and cassava, suggest that small farmers may often receive lower prices where access to markets is limited.

**Sugar yield**	[12]	Cambodia	Annual yield data for large scale plantations from two regions of Cambodia were extracted from ACI[12]. Minimum and maximum reported yields were used as input parameters for the simulation model and were assumed to remain constant over 25 years of repeated annual planting. Yield curves were created for 25 consecutive years of cultivation, as for rubber and cashew, using the same proportion of maximum yield for each year. The run-specific proportion used to create yield curve was generated independently for each crop, for each iteration.
**Sugar production cost**	[12]	Cambodia	Annual production cost data were extracted, standardised to USD ha^{-1} yr^{-1}, adjusted to US$ 2013 prices. Costs included: land clearance (using tractor or bulldozer, year 1 only), land preparation costs (annual), planting material, annual inputs, annual labour costs. Cost curves were created for 25 consecutive years, as for rubber, using the same proportion of maximum cost for each year. The run-specific proportion used to create the cost curve was generated independently for each crop, for each iteration.
Inflating US$ to 2013			
**Consumer price index (CPI)**	[10]	Cambodia/ Thailand/ Vietnam	The World Bank Consumer Price Index (CPI) is available at the country level. For parameters obtained from multi-country studies, CPIs for Cambodia, Thailand and Vietnam were averaged and used to deflate all input costs and prices to 2013.

Supplementary Table 6

Post-deforestation land-use carbon stock estimates. Time-averaged carbon stocks (taCs) of above-ground biomass (AGB) and below-ground biomass (BGB) were estimated as either 50% of the carbon stock of a crop/plantation at the maximum rotation length[26], or for rubber, as the carbon stock as calculated by a regression equation at the median rotation length[27].

Land-use	taCs (tC ha^{-1})	Note
**Rubber**	52.5	Multiple estimates of taCs were generated by Blagodatsky *et al* in a review of studies on rubber plantation carbon dynamics[27]. These estimates were either based on a division of the maximum carbon stock (at the time of clearing) by two, which assumes a linear increase in biomass during the growing cycle, or by fitting a regression model where more detailed data are available, and taking the carbon stock of the plantation as calculated by the equation at the median time in the rotation. Estimates of taCs (AGB + BGB) for 20 – 30 year monoculture rubber plantation cycles in South and Southeast Asia ranged from 40 to 65 tC ha^{-1} [27]; the mean of these values (52.5 tC ha^{-1}) was used in analysis.
**Cashew**	22.32	Out estimate of taCs for a cashew plantation on a 10-year plantation cycle (22.32 tC ha^{-1}) were generated based on field data from Cambodia[28]. AGB of cashew plantations for each year of a 10-year plantation cycle were extracted from the data; BGB was assumed to be 24% of AGB[29], and AGB + BGB carbon stock was assumed to be 50% of biomass. We calculated taCs to be 50% of this value[27]. Where field data were not provided for a given year, the value for the next oldest year was used, generating a conservative estimate.
**Cassava**	2.5	Carbon stock for "annual cropland" in dry and seasonal areas of Asia reported as 5 tC ha^{-1}; time-averaged carbon stock is 50% of this value[26].
**Sugarcane**	6.75	Carbon stock for sugarcane in dry and seasonal areas of Asia reported as 13.5 tC ha^{-1}; time-averaged carbon stock is 50% of this value[26].

Supplementary Table 7

Value of dipterocarp resin collection and influence on breakeven carbon prices

Forest type	Median 25-year resin revenue<br><br>($ ha^{-1})	Median carbon breakeven price: "No timber logged + rubber"<br><br>including resin revenue ($ tCO$_2^{-1}$)	Median carbon breakeven price: "No timber logged + rubber"<br><br>excluding resin revenue ($ tCO$_2^{-1}$)
Dense	357.38	33.43	34.20
Open	234.49	51.12	52.65

Supplementary Table 8

Price estimates for timber royalty classes at various selling points in Cambodia. Minimum and maximum prices for each royalty class for the roadside/village were used as input parameters for simulations; other price points (i.e. forest, domestic or international market) were not used in the final analysis. In the absence of species-specific records from formal timber markets, all timber species in each royalty class were assumed to fetch the same price as those species from that royalty class that were explicitly named in source of prices (Supplementary Table 14). Prices shown are mean of all available data from 2007 - 2014 inclusive, except for Non-Classified timber, for which we use the price of fuelwood reported from field study in Cambodia[30]. All prices were inflated to $US 2013 using a CPI specific to Cambodia. All price data were based on interviews with villagers or market traders.

		Price ($ m^{-3})						
**Price point**	**Royalty Class**	**Min**	**Max**	**Mean Price**	**SD**	**SE**	**n[¥]**	**References**
**Forest**	Luxury	-	-	-	-	-	-	[30,31]
	I	115.4	202.0	152.6	33.1	14.8	5	
	II	116.5	116.5	116.5	-	-	1	
	III	77.7	77.7	77.7	-	-	1	
	NC	17.3	17.3	17.3	-	-	1	
**Roadside/ Village**	Luxury	500.0	3,129.4	1,300.5	657.4	150.8	19	[5,32,33]; Hugh Wright (2010), unpublished data
	I	90.8[#]	290.5	151.7[#]	79.7	28.2	8	
	II	139.7[#]	290.5	251.5	58.6	26.2	5	
	III	77.7*	77.7*	77.7*	-	-	1	
	NC	17.3	17.3	17.3	-	-	1	
**Domestic (national) market**	Luxury	400.0	1154.6	739.3	330.2	147.7	5	[30,31,34–36]
	I	346.3	692.7	517.3	122.8	46.4	7	
	II	461.8	577.3	510.6	40.3	13.4	9	
	III	77.7*	77.7*	77.7*	-	-	1	
	NC	17.3	17.3	17.3	-	-	1	
**International market**	Luxury	3,850.0	50,000.0	18,185.0	16,899.6	5,344.1	10	[36–39]
	I	346.3[˜]	692.7[˜]	517.3[˜]	122.8[˜]	46.4[˜]	7	
	II	461.8[˜]	577.3[˜]	510.6[˜]	40.3[˜]	13.4[˜]	9	
	III	77.7*	77.7*	77.7*	-	-	1	
	NC	17.3	17.3	17.3	-	-	1	

[¥]n price estimates within and across all studies
[#]Royalty class I timber is classed as more valuable than class II[40]; we thus assume the same minimum price for class I and II timber in simulation models ($90.81)
*As for forest price
[˜]As for domestic market price

Supplementary Table 9

Resampling input parameters. For each sampling iteration, values for each parameter were sampled from either a uniform distribution between the minimum and maximum bounds, or where the shape of the distribution was known to be normal, from a normal distribution defined by the mean and standard error (SE) of the mean.

	Input parameter	Units	Bounds
**Agricultural NPV**	Crop input costs (annual)	USD ha^{-1} yr^{-1}	Min – Max
	Farm gate price	USD t^{-1}	Mean – SE
	Crop yield (annual)	t ha^{-1} yr^{-1}	Min – Max
**Carbon stock**	Forest carbon stock (square root transformed)	tC or tCO_2 ha^{-1}	Mean – SE
**Timber profit**	Timber volume (square root transformed)	m^3 ha^{-1}	Mean – SE
	Timber price	USD m^{-3}	Min – Max
	Extraction costs	USD m^{-3}	Min – Max

Supplementary Table 10

Proportion of luxury stems ≥60cm DBH compared between pairs of landscapes by Chi-squared two-sampled proportions test; there were no significant differences.

				F02		F03		F04		F05		F06	
	**Forest type**	**n Luxury stems ≥30cm DBH**	**n Luxury stems ≥60cm DBH**	**X^2**	**p**	**X^2**	**p**	**X^2**	**p**	**X^2**	**p**	**X^2**	**p**
F01	**Dense**	4	0	0.0690	0.79	0.0033	0.95	0.0000	1.00	0.0000	1.00	0.2344	0.63
F02	**Dense**	111	20	-	-	0.0000	1.00	2.1100	0.15	0.0047	0.95	0.1591	0.69
F03	**Dense**	11	2	-	-		-	0.4052	0.52	0.0000	1.00	0.0111	0.92
F04	**Dense**	34	2	-	-	-	-	-	-	0.0000	1.00	1.7647	0.18
F05	**Dense**	9	1	-	-	-	-	-	-	-	-	0.1563	0.69
F06	**Dense**	6	2	-	-	-	-	-	-	-	-	-	

				**F02**		**F04**	
	**Forest type**	**n Luxury stems ≥30cm DBH**	**n Luxury stems >=60cm DBH**	**X^2**	**p**	**X^2**	**p**
F01	**Open**	6	1	0.0000	1.00	0.0000	1.00
F02	**Open**	21	4	-	-	0.2431	0.62
F04	**Open**	14	1	-	-	-	-

Supplementary Table 11

Input parameters for agricultural net present value calculation. Year 1 includes land clearance costs entailed in preparing logged-over land for agriculture; $450 for large plantations (mechanised, bulldozer) or $250 for smallholders (manual labour; [12]). See Supplementary Table 5 for data sources. All values in 2013 USD.

Crop	Size	Year	Annual cost ($ ha^{-1} yr^{-1}): minimum	Annual cost ($ ha^{-1}yr^{-1}): maximum	Farmgate price ($ t^{-1}) mean	Farmgate price ($ t^{-1}) SE	Annual yield – closed forest (t ha^{-1} yr^{-1}): minimum	Annual yield – closed forest (t ha^{-1} yr^{-1}): maximum	Annual yield – open forest (t ha^{-1} yr^{-1}): minimum	Annual yield – open forest (t ha^{-1} yr^{-1}): maximum
**Cashew**	Smallholder	0	514.06	764.61	963.48	43.75	0.00	0.00	0.00	0.00
		1	31.42	125.69	963.48	43.75	0.00	0.00	0.00	0.00
		2	27.23	173.88	963.48	43.75	0.00	1.90	0.00	1.90
		3	27.23	173.88	963.48	43.75	0.30	1.90	0.30	1.90
		4	27.23	173.88	963.48	43.75	0.30	1.90	0.30	1.90
		5	54.47	167.59	963.48	43.75	0.60	2.30	0.60	2.30
		6	54.47	167.59	963.48	43.75	0.70	2.30	0.70	2.30
		7	54.47	167.59	963.48	43.75	0.77	2.30	0.77	2.30
		8	54.47	167.59	963.48	43.75	0.77	2.30	0.77	2.30
		9	54.47	167.59	963.48	43.75	0.77	2.30	0.77	2.30
		10	54.47	173.88	963.48	43.75	0.77	3.00	0.77	3.00
		11	54.47	173.88	963.48	43.75	0.77	3.00	0.77	3.00
		12	54.47	173.88	963.48	43.75	0.77	3.00	0.77	3.00
		13	54.47	173.88	963.48	43.75	0.77	3.00	0.77	3.00
		14	54.47	173.88	963.48	43.75	0.77	3.00	0.77	3.00
		15	54.47	173.88	963.48	43.75	0.60	2.31	0.60	2.31
		16	54.47	173.88	963.48	43.75	0.60	2.31	0.60	2.31
		17	54.47	173.88	963.48	43.75	0.60	2.31	0.60	2.31
		18	54.47	173.88	963.48	43.75	0.60	2.31	0.60	2.31
		19	54.47	173.88	963.48	43.75	0.60	2.31	0.60	2.31
		20	54.47	173.88	963.48	43.75	0.60	2.31	0.60	2.31
		21	54.47	173.88	963.48	43.75	0.60	2.31	0.60	2.31
		22	54.47	173.88	963.48	43.75	0.60	2.31	0.60	2.31
		23	54.47	173.88	963.48	43.75	0.60	2.31	0.60	2.31
		24	54.47	173.88	963.48	43.75	0.60	2.31	0.60	2.31
**Cassava**	Smallholder	0	412.25	860.90	90.51	6.22	10.50	30.10	10.50	30.10
		1	110.29	560.00	90.51	6.22	10.50	30.10	10.50	30.10
		2	110.29	560.00	90.51	6.22	10.50	30.10	10.50	30.10
		3	110.29	560.00	90.51	6.22	10.50	30.10	10.50	30.10
		4	110.29	560.00	90.51	6.22	10.50	30.10	10.50	30.10
		5	110.29	560.00	90.51	6.22	10.50	30.10	10.50	30.10
		6	110.29	560.00	90.51	6.22	10.50	30.10	10.50	30.10
		7	110.29	560.00	90.51	6.22	10.50	30.10	10.50	30.10
		8	110.29	560.00	90.51	6.22	10.50	30.10	10.50	30.10
		9	110.29	560.00	90.51	6.22	10.50	30.10	10.50	30.10
		10	110.29	560.00	90.51	6.22	10.50	30.10	10.50	30.10
		11	110.29	560.00	90.51	6.22	10.50	30.10	10.50	30.10
		12	110.29	560.00	90.51	6.22	10.50	30.10	10.50	30.10
		13	110.29	560.00	90.51	6.22	10.50	30.10	10.50	30.10
		14	110.29	560.00	90.51	6.22	10.50	30.10	10.50	30.10
		15	110.29	560.00	90.51	6.22	10.50	30.10	10.50	30.10
		16	110.29	560.00	90.51	6.22	10.50	30.10	10.50	30.10
		17	110.29	560.00	90.51	6.22	10.50	30.10	10.50	30.10
		18	110.29	560.00	90.51	6.22	10.50	30.10	10.50	30.10
		19	110.29	560.00	90.51	6.22	10.50	30.10	10.50	30.10
		20	110.29	560.00	90.51	6.22	10.50	30.10	10.50	30.10
		21	110.29	560.00	90.51	6.22	10.50	30.10	10.50	30.10
		22	110.29	560.00	90.51	6.22	10.50	30.10	10.50	30.10
		23	110.29	560.00	90.51	6.22	10.50	30.10	10.50	30.10
		24	110.29	560.00	90.51	6.22	10.50	30.10	10.50	30.10

**Sugar**	Large plantation	0	1168.20	1749.82	35.74	1.30	12.00	29.50	12.00	29.50
		1	586.58	586.58	35.74	1.30	12.00	29.50	12.00	29.50
		2	586.58	586.58	35.74	1.30	12.00	29.50	12.00	29.50
		3	586.58	586.58	35.74	1.30	12.00	29.50	12.00	29.50
		4	586.58	586.58	35.74	1.30	12.00	29.50	12.00	29.50
		5	586.58	586.58	35.74	1.30	12.00	29.50	12.00	29.50
		6	586.58	586.58	35.74	1.30	12.00	29.50	12.00	29.50
		7	586.58	586.58	35.74	1.30	12.00	29.50	12.00	29.50
		8	586.58	586.58	35.74	1.30	12.00	29.50	12.00	29.50
		9	586.58	586.58	35.74	1.30	12.00	29.50	12.00	29.50
		10	586.58	586.58	35.74	1.30	12.00	29.50	12.00	29.50
		11	586.58	586.58	35.74	1.30	12.00	29.50	12.00	29.50
		12	586.58	586.58	35.74	1.30	12.00	29.50	12.00	29.50
		13	586.58	586.58	35.74	1.30	12.00	29.50	12.00	29.50
		14	586.58	586.58	35.74	1.30	12.00	29.50	12.00	29.50
		15	586.58	586.58	35.74	1.30	12.00	29.50	12.00	29.50
		16	586.58	586.58	35.74	1.30	12.00	29.50	12.00	29.50
		17	586.58	586.58	35.74	1.30	12.00	29.50	12.00	29.50
		18	586.58	586.58	35.74	1.30	12.00	29.50	12.00	29.50
		19	586.58	586.58	35.74	1.30	12.00	29.50	12.00	29.50
		20	586.58	586.58	35.74	1.30	12.00	29.50	12.00	29.50
		21	586.58	586.58	35.74	1.30	12.00	29.50	12.00	29.50
		22	586.58	586.58	35.74	1.30	12.00	29.50	12.00	29.50
		23	586.58	586.58	35.74	1.30	12.00	29.50	12.00	29.50
		24	586.58	586.58	35.74	1.30	12.00	29.50	12.00	29.50
**Rubber**	Large plantation	0	1141.38	1902.64	2595.56	200.27	0.00	0.00	0.00	0.00
		1	174.40	289.94	2595.56	200.27	0.00	0.00	0.00	0.00
		2	79.61	424.01	2595.56	200.27	0.00	0.00	0.00	0.00
		3	79.61	447.47	2595.56	200.27	0.00	0.00	0.00	0.00
		4	79.61	447.47	2595.56	200.27	0.00	0.00	0.00	0.00
		5	349.20	447.47	2595.56	200.27	0.00	1.36	0.00	0.00
		6	349.20	1169.80	2595.56	200.27	0.24	1.36	0.00	0.00
		7	349.20	1178.18	2595.56	200.27	0.50	1.36	0.00	0.00
		8	349.20	1178.18	2595.56	200.27	0.50	1.50	0.00	0.00
		9	349.20	1178.18	2595.56	200.27	0.50	1.50	0.00	1.36
		10	333.35	1178.18	2595.56	200.27	0.99	2.00	0.24	1.36
		11	333.35	1178.18	2595.56	200.27	0.99	2.00	0.50	1.36
		12	333.35	1178.18	2595.56	200.27	0.99	2.00	0.50	1.50
		13	333.35	1178.18	2595.56	200.27	0.99	2.00	0.50	1.50
		14	333.35	1178.18	2595.56	200.27	0.99	2.00	0.99	2.00
		15	322.30	1178.18	2595.56	200.27	1.06	2.25	0.99	2.00
		16	322.30	1178.18	2595.56	200.27	1.06	2.25	0.99	2.00
		17	322.30	1178.18	2595.56	200.27	1.06	2.30	0.99	2.00
		18	322.30	1178.18	2595.56	200.27	1.06	2.30	0.99	2.00
		19	322.30	1178.18	2595.56	200.27	1.06	2.30	1.06	2.25
		20	322.30	1178.18	2595.56	200.27	1.06	2.30	1.06	2.25
		21	322.30	1178.18	2595.56	200.27	1.06	2.30	1.06	2.30
		22	322.30	1178.18	2595.56	200.27	1.06	2.30	1.06	2.30
		23	322.30	1178.18	2595.56	200.27	1.06	2.30	1.06	2.30
		24	322.30	1178.18	2595.56	200.27	1.06	2.30	1.06	2.30

Supplementary Table 12

Harvestable timber volume equations for evergreen, mixed and deciduous forests. Timber volume equations, that estimate harvestable volume rather than tree volume, were obtained from the Forestry Administration of the Royal Government of Cambodia[41]**.** For each tree, timber volume was calculated from diameter at breast height (*DBH*, in m) using the equation appropriate to size class, tree type (dipterocarp or non-dipterocarp; unknown species assumed to be non-dipterocarp) and forest type. The deciduous forest equation was used for all open forest plots; for dense forest plots the evergreen equation was used as it gave consistently lower volume estimates than mixed forest equations, thereby making estimated timber volumes conservative. Although additional equations were available that incorporate tree height (*H*, in m), forest inventories did not provide height estimates and region-specific form factors (required to estimate height from DBH data[42]) were not available for Cambodia; therefore, DBH-only equations were used. Tree volumes (m^3) were summed per plot and per royalty class and standardised to m^3 ha^{-1}. Final harvestable timber volume was reduced by 20% to account for wastage[43].

Forest type	Tree type	DBH	Equation
**Evergreen**	Dipterocarp	<15cm	Volume (m^3) = 0.022 + 3.4*DBH^2
	Dipterocarp	≥15 cm	Volume (m^3) = -0.0971 + 9.503*DBH^2
	Non-Dipterocarp	<30 cm	Volume (m^3) = 0.03 + 2.8*DBH^2
	Non-Dipterocarp	≥30 cm	Volume (m^3) = -0.331 + 6.694*DBH^2
**Mixed**	Dipterocarp	<15cm	Volume (m^3) = 0.03 + 4.8 * DBH^2
	Dipterocarp	≥15 cm	Volume (m^3) = 0.00126 +6.167 DBH^2
	Non-Dipterocarp	<15 cm	Volume (m^3) = 0.0083 + 4.3 DBH^2
	Non-Dipterocarp	15-30 cm	Volume (m^3) = 0.0083 + 5.3 DBH^2
	Non-Dipterocarp	≥30 cm	Volume (m^3) = 0.0083 + 6.081 DBH^2
**Deciduous**	Dipterocarp	<15cm	Volume (m^3) = 0.00849 + 4.097 * DBH^2
	Dipterocarp	≥15 cm	Volume (m^3) = -0.051 + 5.864 * DBH^2
	Non-Dipterocarp	<15 cm	Volume (m^3) = 0.03 + 3.3 DBH^2
	Non-Dipterocarp	15-30 cm	Volume (m^3) = 0.03 + 3.55 DBH^2
	Non-Dipterocarp	≥30 cm	Volume (m^3) = -0.413 + 7.819 DBH^2

Supplementary Table 13

Timber species named in roadside/village price estimates. These prices were applied to all species in the same royalty class as the named species i.e. all Class I species were given the same price, based on price estimates for *Lagerstroemia sp*, *Hopea odorata*, *Sindora siamensis*, *Xylia dolabriformis* and *Tarrietia javanica*.

Royalty Class	Species
**I**	*Lagerstroemia sp*
	Hopea odorata
	Sindora siamensis
	Xylia dolabriformis
	Tarrietia javanica
**II**	*Anisoptera sp*
	Dipterocarpus sp
	Dipterocarpus alatus
	Dipterocarpus tuberculatus
	Dipterocarpus obtusifolius
**III**	Unknown*
**Luxury**	*Afzelia cochinchinensis*
	Dalbergia oliveri/bariensis#
	Pterocarpus pedatus
	Dalbergia cochinchinensis/D. bariensis#
**NC**	Unknown¥

¥one price given for fuelwood, applied to all NC timbers[1]
#*Dalbergia bariensis* is a synonym of *D. oliveri* but is commonly referred to as *D. bariensis* in Cambodia[44]; *D. cochinchinesis* is listed on CITES Appendix II
*one price given for all class III timbers, at forest price point[31]

Supplementary Table 14

Timber extraction cost estimates. The minimum and maximum timber extraction costs from this table were used as input parameters for simulating timber costs. These costings assume selective logging activity by local people in Cambodia in a 'business-as-usual' scenario with no formal logging concessions, inventories, management plan, or demarcation of logging areas. Costs include: wage labour, food, motorbike fuel, ox-cart transportation to the roadside/village and chainsaw maintenance but exclude the capital cost of the chainsaw (around US$350[30]). Costs in table are inflated to US$2013 using a CPI specific to Cambodia.

Reference	Extraction Cost USD m^{-3} processed wood	Notes
[30]	116.01	Cost of cutting wood in forest and ox-cart transport to village. Labour, food, fuel, chainsaw oil, 2-stroke oil, chain, chainsaw maintenance, excludes capital cost of chainsaw ($350 dollars, last 10 years), ox cart to village.
[31]	75.77	Cost of cutting wood in forest and ox-cart transport to village. Hired labour to cut tree, chainsaw fuel, ox cart to village.
[1]	82.36	Cost of partial cut and transport (to village).

Supplementary Table 15

Dipterocarpus spp. resin revenue estimation parameters.

Parameter	Reference	Notes
**Resin yield per tree per year**	[45–47]	23 – 40 litres yr^{-1} reported as maximum and minimum yields, across all three studies, across tree species
**Tree sizes tapped for resin**	[45]	Trees 40 – 50cm DBH and upwards are preferred, but trees as small as 30cm DBH can be tapped
**Non yielding trees (non-starter or exhausted)**	[45]	Of 2,555 trees surveyed, 62 (2.4%) were non-starters and 146 (5.7%) were exhausted; thus only 208 (8.1%) were non-yielding
**Resin price**	[45,46,48] and WCS, unpublished data.	Price in US$ $litre^{-1}$; mean of estimates (adjusted to 2013 US$) from 2003 – 2014 was $0.37 per litre

Supplementary Note 1

Forest degradation through logging has a complex history in Cambodia. Forest governance institutions were lost during the political turmoil of the Khmer Rouge era (1975 – 1980). Subsequently, nearly 70% of forested land was allocated for logging concessions in the 1990s, followed by widespread over-harvesting both within and outside concessions[49]. All formal logging concessions were halted in 2002, and many have since been designated as protected areas. Forested land is owned by the state and some annual logging coupes have been allocated; however, large tracts of forest have no clear management plan and illegal logging remains pervasive[36,38,49–51]. Allocation of forested areas for Economic Land Concessions (ELCs), which allow conversion to plantation crops, is a key driver of forest clearance in Cambodia; much of Cambodia's current timber harvest is extracted within and around ELCs[52]. Much focus has been placed on the extraction of the highest-value Luxury class timber[50], which can generate high levels of short-term income[33]. However, logging of other species (of lower royalty classes, especially classes I & II) is also pervasive[5] and forms the bulk of timber harvested when forested land is cleared from ELCs[53].

The Forestry Administration grants transport licenses for logs ≥30cm DBH harvested from within ELCs, except for luxury timber[53]. Minimum harvestable limits for all commercial tree species range from 30 – 60cm DBH (as defined by the Ministry of Agriculture, Forestry and Fisheries in Prakas 089 (2005) – a ministerial or inter-ministerial proclamation in Cambodian law), however the level of enforcement of these limits is not clear. According to a Ministerial Declaration, Prakas 089, harvest of all luxury class timber is illegal, as is harvest of resin trees (some *Dipterocarpus* spp, all royalty class II, Supplementary Table 2) utilised by local people, unless they have given consent and been compensated. This latter group includes some of the most commercially valuable dipterocarp species. However, there is evidence for routine and widespread harvest of both luxury and resin trees[54]. In the 1990s commercial logging focussed on trees ≥45cm DBH[55,56], while minimum commercial harvestable DBH elsewhere in Southeast Asia is ≥40cm DBH[57], and an assessment of logging in Cambodia that modelled unsustainable extraction rates assumed trees ≥40cm would be harvested[58]. For class I and II species, we therefore assumed a minimum harvestable DBH of 40 cm. Luxury species are exceptionally valuable and even small amounts are harvested[59]; we therefore assumed all luxury class trees ≥10cm DBH would be harvested. Class III trees are used for local construction purposes (i.e. as timber) or as fuelwood. Non-classified trees are assumed to be only useful as fuelwood; non-classified and class III trees ≥40cm DBH were therefore assumed to have market value as fuelwood.

Supplementary Note 2

Cassava (163% area increase 2009 – 2013, to 421,000 ha[60]), sugarcane (76% area increase 2009 – 2013, to 23,810 ha[60]) and cashew (16,000 ha in 2000, 60,000 ha in 2005, no recent data available[13]) are also rapidly expanding cash crops. In Cambodia, cash-crops may be grown by smallholders (typically cassava, cashew and some rubber in farms of approximately 1-50 ha in size) or by concessionaires in large agro-industrial plantations (typically rubber or sugar).

Dipterocarp resin collection is a traditional livelihood activity that generates important cash income, which directly conflicts with logging, as resin-producing species have valuable timber[1,45,61]. Other local benefits derived from forests, including fuelwood and bushmeat[1,56,62], could not be estimated on a per hectare basis, as they depend on household density, extraction rates and the cost of substitute resources in local markets. However, non-market environmental income can contribute 32 – 35% of household income, of which 70% comes from forest products, excluding resin revenues[62]. As resin collection contributes only a portion of forest product income[1], a substantial proportion of the value of standing forests to local people is not captured in our analysis.

Supplementary references

1. Hansen, K. K. & Neth, T. *Natural forest benefits and economic analysis of natural forest conversion in Cambodia. Working Paper 33.* (Cambodia Development Resource Institute, 2006).
2. Lambrick, F. H., Brown, N. D., Lawrence, A. & Bebber, D. P. Effectiveness of community forestry in Prey Long Forest, Cambodia. *Conserv. Biol.* **28,** 372–381 (2014).
3. Theilade, I., Schmidt, L., Chhang, P. & McDonald, J. A. Evergreen swamp forest in Cambodia: floristic composition, ecological characteristics, and conservation status. *Nord. J. Bot.* **29,** 71–80 (2011).
4. Sawada, H., Araki, M., Chappell, N. A., LaFrankie, J. V & Shimizu, A. *Forest Environments in the Mekong River Basin*. (Springer, 2007).
5. WCS. *Reduced Emissions from Deforestation and Degradation in Seima Protection Forest, Cambodia. Project Description.* (Wildlife Conservation Society for the Forestry Administration of the Royal Government of Cambodia, 2015).
6. Gilroy, J. J. *et al.* Cheap carbon and biodiversity co-benefits from forest regeneration in a hotspot of endemism. *Nat. Clim. Chang.* **4,** 503–507 (2014).
7. Goldstein, A. *Converging at the crossroads. State of forest carbon finance 2015*. (Forest Trends' Ecosystem Marketplace, 2015).
8. Goldstein, A. *Buying in: taking stock of the role of offsets in corporate carbon strategies*. (Forest Trends' Ecosystem Marketplace, 2016).
9. Cama, T. Court backs Obama's climate change accounting. *The Hill* (2016).
10. The World Bank. The World Bank. Consumer price index (2010 = 100). (2015). at <http://data.worldbank.org/indicator/FP.CPI.TOTL>
11. FAO. FAOSTAT Online Statistical Service. (2014). at <http://faostat.fao.org>
12. ACI. *Final Report for the Cambodian Agrarian Structure Study. Prepared for the Ministry of Agriculture, Forestry and Fisheries, Royal Government of Cambodia, the World Bank, the Canadian International Development Agency (CIDA) and the Government of Germany/Ges*. (Agrifood Consulting International, 2005).
13. EIC. *Export Diversification and Value Addition for Human Development*. (Economic Institute of Cambodia, 2007).
14. Hing, V. & Thun, V. *Agricultural trade in the Greater Mekong sub-region: the case of cassava and rubber in Cambodia. Working paper series No. 43.* (CDRI, 2009).
15. Shigematsu, A. *et al.* Financial potential of rubber plantations considering rubberwood production: wood and crop production nexus. *Biomass Bioenergy* **49,** 131–142 (2013).
16. Carr, M. K. V. The water relations of rubber (Hevea brasiliensis): a review. *Exp. Agric.* **48,** 176–193 (2012).
17. IFC. *Prospects for Cambodia's cashew sub-sector*. (International Finance Corporation, World Bank Group, 2010).
18. AFSIS. AFSIS ASEAN Food Security Information System Statistics. Cambodia. Table 5. CASSAVA: Planted Area, Harvested Area, Production and Yield - Cambodia. (2016). at <http://www.afsisnc.org/statistics/data-selected?view=result&tbl_type=5&tbl_id=Cassava&cty_id=Cambodia>
19. Agricultural Marketing Information Service. Agricultural Market Information. Commodity Price by Market. (2016). at <http://www.agriculturalmarketinformation.org.kh/en/price-and-production-data/price-data/commodity-price-by-market>
20. SNV Cambodia. *Cassava value chain analysis. Inclusive business model for promoting sustainable smallholder cassava production (IBC)*. (SNV Netherlands Development Organisation, 2015).
21. Sopheap, U., Patanothai, A. & Aye, T. M. Unveiling constraints to cassava production in Cambodia: An analysis from farmers' yield variations. *Int. J. Plant Prod.* **6,** 409–428 (2012).
22. Sopheap, U., Patanothai, A. & Aye, T. M. Farmers' perceptions on cassava cultivation in Cambodia. *Khon Kaen*

Agric. J. 39: 279-294 (2011).

23. Howeler, R. H. H. Long-term effect of cassava cultivation on soil productivity. *F. Crop. Res.* **26,** 1–18 (1991).

24. Van Eijck, J., Smeets, E. & Faaij, A. The economic performance of jatropha, cassava and Eucalyptus production systems for energy in an East African smallholder setting. *GCB Bioenergy* **4,** 828–845 (2012).

25. Equitable Cambodia & Inclusive Development International. *Bittersweet harvest: a human rights impact assessment of the European Union's Everything But Arms initiative in Cambodia*. (Equitable Cambodia, Inclusive Development International, 2013).

26. Gibbs, H. K. *et al.* Carbon payback times for crop-based biofuel expansion in the tropics: the effects of changing yield and technology. *Environ. Res. Lett.* **3,** 34001 (2008).

27. Blagodatsky, S., Xu, J. & Cadisch, G. Carbon balance of rubber (Hevea brasiliensis) plantations: a review of uncertainties at plot, landscape and production level. *Agric. Ecosyst. Environ.* **221,** 8–19 (2016).

28. Avtar, R., Takeuchi, W. & Sawada, H. Monitoring of biophysical parameters of cashew plants in Cambodia using ALOS/PALSAR data. *Environ. Monit. Assess.* **185,** 2023–2037 (2013).

29. Cairns, M. A., Brown, S., Helmer, E. H. & Baumgardner, G. A. Root biomass allocation in the world's upland forests. *Oecologia* **111,** 1–11 (1997).

30. Blackett, H. *A study of the Cambodia timber trade: Market analysis for the Commercial Community Forestry Project*. (Forestry Administration; Wildlife Conservation Society, 2008).

31. Grimm, J., Evans, T., Mesa, H. & Ratanakoma, L. *Commercial community forestry in Cambodia. Development of a pilot project in the Seima Biodiversity Conservation Area.* (Wildlife Conservation Society Cambodia Programme, 2007).

32. Titthara, M. Caught up in the middle. 8 September 2014. *The Phnom Penh Post* (2014).

33. Singh, S. The socio-economic context of illegal logging and trade of rosewood along the Cambodian-Lao border. *For. Trends Rep. Ser.* 9 (2013).

34. Seangly, P. *Forest razed to tune of $100 million. 25 April 2013. The Phnom Penh Post* (2013).

35. Pye, D. *Gov't releases data on timber baron's deal. 19 September 2014*. *The Phnom Penh Post* (2014).

36. Peter, Z. & Pheap, A. *How Cambodia's secretive timber auctions are fueling the illegal logging trade. 14 July 2014. The Cambodia Daily* (2014).

37. EIA. *Appetite for destruction*. (Environmental Investigation Agency, 2012).

38. Pye, D. & Titthara, M. *The calculus of logging. 10 October 2014. The Phnom Penh Post* (2014).

39. Pye, D. *Furnishing a bad habit. 13 May 2014. The Phnom Penh Post* (2014).

40. FA. *Forest systems research and modelling handbook regional volume table. Appendix 1 - list of species and respective species groups*. (Kingdom of Cambodia, Forestry Administration; Indufor Oy; Foret Ressources Management; Societe Generale de Surveillance, 2004).

41. FA. *Forest Concession Management and Control Pilot Project. Document 3 - Regional Volume Table*. (Kingdom of Cambodia, Forestry Administration; Indufor Oy; Foret Ressources Management; Societe Generale de Surveillance, 2004).

42. Feldpausch, T. R. *et al.* Height-diameter allometry of tropical forest trees. *Biogeosciences* **8,** 1081–1106 (2011).

43. Putz, F. E., Sist, P., Fredericksen, T. & Dykstra, D. Reduced-impact logging: challenges and opportunities. *For. Ecol. Manage.* **256,** 1427–1433 (2008).

44. Hartvig, I., Czako, M., Kjær, E. D., Nielsen, L. R. & Theilade, I. The use of DNA barcoding in identification and conservation of rosewood (Dalbergia spp.). *PLoS One* **10,** (2015).

45. Evans, T. D., Piseth, H., Phaktra, P. & Mary, H. *A study of resin-tapping and livelihoods in Southern Mondulkiri, Cambodia, with implications for conservation and forest management*. (Wildlife Conservation Society, 2003).

46. Tola, P. & McKenney, B. *Trading forest products in Cambodia: challenges, threats and pppportunities for resin - Working Paper 28.* (Cambodia Development Resource Institute, 2003).

47. Orwa, C., Mutua, A., Kindt, R., Jamnadass, R. & Anthony, S. Agroforestree Database: a tree reference and selection guide version 4.0. (2009). at <http://www.worldagroforestry.org/resources/databases/agroforestree>

48. Tola, P. *Beyond subsistence. Trade chain analysis of resin products in Cambodia*. (NTFP Exchange Programme for South and Southeast Asia and the Cambodia NTFP Working Group, 2009).

49. Blaser, J., Sarre, A., Poore, D. & Johnson, S. *Status of Tropical Forest Management 2011. ITTO Technical Series No. 38* (International Tropical Timber Organisation, 2011).

50. EIA. *Routes of extinction: The corruption and violence destroying Siamese rosewood in the Mekong*. (Environmental Investigation Agency, 2014).

51. Milne, S. Cambodia's unofficial regime of extraction: illicit logging in the shadow of transnational governance and investment. *Crit. Asian Stud.* **47,** 200–228 (2015).

52. Forest Trends. *Conversion Timber, Forest Monitoring, and Land-Use Governance in Cambodia*. *Forest Trends Report Series* (Forest Trends, 2015).

53. TWGFA, FA & EU FLEGT. *Understanding timber flows and control in Cambodia in the context of FLEGT Prepared by Global Forestry Services*. (Global Forestry Services; Forestry Administration of Cambodia; EU FLEGT, 2014).

54. Global Witness. *The cost of luxury*. (Global Witness, 2015).

55. Kao, D. & Iida, S. Structural characteristics of logged evergreen forests in Preah Vihear, Cambodia, 3 years after logging. *For. Ecol. Manage.* **225,** 62–73 (2006).

56. De Lopez, T. T. Economics and stakeholders of Ream National Park, Cambodia. *Ecol. Econ.* **46,** 269–282 (2003).

57. Fisher, B. *et al.* Cost-effective conservation: calculating biodiversity and logging trade-offs in Southeast Asia. *Conserv. Lett.* **4,** 443–450 (2011).

58. McKenney, B., Chea, Y., Tola, P. & Evans, T. *Focusing on Cambodia's high value forests: livelihoods and management*. (Cambodia Development Resource Institute; Wildlife Conservation Society, 2004).

59. EIA. *Rosewood Robbery. The Case for Thailand to List Rosewood on CITES*. (Environmental Investigation Agency, 2012).

60. MAFF. *Agricultural Sector Strategic Development Plan 2014-2018*. (Ministry of Agriculture, Forestry and Fisheries, Royal Government of Cambodia, 2015).

61. Theilade, I. & Schmidt, L. H. *REDD+ and conservation of Prey Long Forest, Cambodia: summary of scientific findings 2007-2010*. *Working Papers Forest & Landscape No. 66/2011* (Forest & Landscape, University of Copenhagen, 2011).

62. Jiao, X., Smith-Hall, C. & Theilade, I. Rural household incomes and land grabbing in Cambodia. *Land Use Policy* **48,** 317–328 (2015).

63. IUCN. The IUCN Red List of Threatened Species. Version 2014.3. (2014). at <http://www.iucnredlist.org>

64. Kim, S., Phat, N., Koike, M. & Hayashi, H. Estimating actual and potential government revenues from timber harvesting in Cambodia. *For. Policy Econ.* **8,** 625–635 (2006).

65. The Plant List. Version 1.1. (2013). at <http://www.theplantlist.org>

66. Appanah, S., Turnbull, J. M. & Research, C. I. F. *A Review of Dipterocarps: Taxonomy, Ecology, and Silviculture*. (Center for International Forestry Research, 1998).

67. Baird, I. G. & Dearden, P. Biodiversity conservation and resource tenure regimes: A case study from Northeast Cambodia. *Environ. Manage.* **32,** 541–550 (2003).

68. Conservation International. *Prey Lang Forest Landscape. A Biodiversity Assessment for Forestry Administration by Conservation International & Winrock International*. (Conservation International; Winrock International, 2015).

69. Huljus, J. & Jell, B. *Cambodia. Review of strategic forest management plans prepared by concession companies operating in Cambodia. Part II. Final report.* (GFA Terra Systems, 2005).

70. Sok Tha, M. & Aare Olsen, J. *Cambodia Tree Seed Project / Danida. National Priority Tree Species Workshop, Phnom Penh, 15 - 16 August 2000*. (Cambodia Tree Seed Centre, Department of Forestry and Wildlife, Ministry of Agriculture, Forestry and Fisheries, 2001).
