# Supplementary material for: Protecting tropical forests from the rapid expansion of rubber using carbon payments
Source: Nat Commun. 2018 Mar 2;9:911. doi: 10.1038/s41467-018-03287-9 (PMC5834519; doi:10.1038/s41467-018-03287-9)
Supplement: Supplementary file 2 — Description of Additional Supplementary Files [file 41467_2018_3287_MOESM2_ESM.pdf]

Description of Additional Supplementary Files

File Name: Supplementary Data 1
Description: **Commercial timber species and royalty classes in Cambodia.**

Timber royalty classes published by the Forestry Administration of Cambodia[40], with IUCN threat category[63]. Royalty classes determine the royalty payment that should be paid to the Royal Government of Cambodia when felling trees[64] and reflect commercial value[1]. Luxury class timber commands the highest commercial value (see Supplementary Table 8) and royalty payment, followed in descending order by I, II, III and non-classified (NC) species. When assigning tree species from forest inventories to timber royalty classes, both Khmer and scientific species names in inventories were checked against the timber royalty class list; trees without species names and species recorded in forest inventories not listed on the FA timber royalty class list[40] were classed as NC, except where specified in notes a - f. Species listed on the royalty class list but not recorded in any of the forest inventories are listed here for reference. Nomenculature in column "Scientific name" follows The Plant List[65]. Scientific names as recorded in the forest inventories or the timber royalty class list[40] are also shown, in column "Recorded scientific name". Mean density (and 95% confidence interval) of stems ≥10 cm DBH in dense and open forest were calculated from plot-level data for each inventory. Inventory names have been anonymised in the interest of data confidentiality and security for sites containing high value timber. References cited here can be found at the end of the Supplementary Information file.
